# Glucose-lowering effects of a synbiotic combination containing *Pediococcus acidilactici* in *C. elegans* and mice

**DOI:** 10.1007/s00125-023-05981-w

**Published:** 2023-08-16

**Authors:** Deyan Yavorov-Dayliev, Fermín I. Milagro, Josune Ayo, María Oneca, Ignacio Goyache, Miguel López-Yoldi, Paula Aranaz

**Affiliations:** 1 Genbioma Aplicaciones SL, Navarra, Spain; 2 Fac Pharm & Nutr, Dept Nutr Food Sci & Physiol, University of Navarra, Pamplona, Spain; 3 Center for Nutrition Research, University of Navarra, Pamplona, Spain; 4 Navarra Institute for Health Research (IdiSNA), Pamplona, Spain; 5 Centro de Investigación Biomédica en Red de la Fisiopatología de la Obesidad y Nutrición (CIBERObn), Instituto de Salud Carlos III, Madrid, Spain

**Keywords:** Diabetes, Microbiota, Obesity, Probiotics, Synbiotic, The metabolic syndrome

## Abstract

**Aims/hypothesis:**

Modulation of gut microbiota has emerged as a promising strategy to treat or prevent the development of different metabolic diseases, including type 2 diabetes and obesity. Previous data from our group suggest that the strain *Pediococcus acidilactici* CECT9879 (pA1c) could be an effective probiotic for regulating glucose metabolism. Hence, the objectives of this study were to verify the effectiveness of pA1c on glycaemic regulation in diet-induced obese mice and to evaluate whether the combination of pA1c with other normoglycaemic ingredients, such as chromium picolinate (PC) and oat β-glucans (BGC), could increase the efficacy of this probiotic on the regulation of glucose and lipid metabolism.

**Methods:**

*Caenorhabditis elegans* was used as a screening model to describe the potential synbiotic activities, together with the underlying mechanisms of action. In addition, 4-week-old male C57BL/6J mice were fed with a high-fat/high-sucrose diet (HFS) for 6 weeks to induce hyperglycaemia and obesity. Mice were then divided into eight groups (*n*=12 mice/group) according to dietary supplementation: control-diet group; HFS group; pA1c group (10^10^ colony-forming units/day); PC; BGC; pA1c+PC+BGC; pA1c+PC; and pA1c+BGC. Supplementations were maintained for 10 weeks. Fasting blood glucose was determined and an IPGTT was performed prior to euthanasia. Fat depots, liver and other organs were weighed, and serum biochemical variables were analysed. Gene expression analyses were conducted by real-time quantitative PCR. Sequencing of the V3–V4 region of the 16S rRNA gene from faecal samples of each group was performed, and differential abundance for family, genera and species was analysed by ALDEx2R package.

**Results:**

Supplementation with the synbiotic (pA1c+PC+BGC) counteracted the effect of the high glucose by modulating the insulin–IGF-1 signalling pathway in *C. elegans*, through the reversal of the glucose nuclear localisation of *daf-16*. In diet-induced obese mice, all groups supplemented with the probiotic significantly ameliorated glucose tolerance after an IPGTT, demonstrating the glycaemia-regulating effect of pA1c. Further, mice supplemented with pA1c+PC+BGC exhibited lower fasting blood glucose, a reduced proportion of visceral adiposity and a higher proportion of muscle tissue, together with an improvement in the brown adipose tissue in comparison with the HFS group. Besides, the effect of the HFS diet on steatosis and liver damage was normalised by the synbiotic. Gene expression analyses demonstrated that the synbiotic activity was mediated not only by modulation of the insulin–IGF-1 signalling pathway, through the overexpression of GLUT-1 and GLUT-4 mediators, but also by a decreased expression of proinflammatory cytokines such as monocyte chemotactic protein-1. 16S metagenomics demonstrated that the synbiotic combinations allowed an increase in the concentration of *P. acidilactici*, together with improvements in the intestinal microbiota such as a reduction in *Prevotella* and an increase in *Akkermansia muciniphila*.

**Conclusions/interpretation:**

Our data suggest that the combination of pA1c with PC and BGC could be a potential synbiotic for blood glucose regulation and may help to fight insulin resistance, diabetes and obesity.

**Graphical Abstract:**

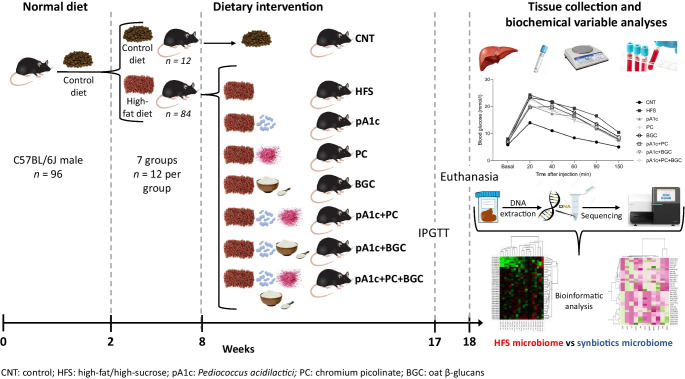

**Supplementary Information:**

The online version contains peer-reviewed but unedited supplementary material available at 10.1007/s00125-023-05981-w.


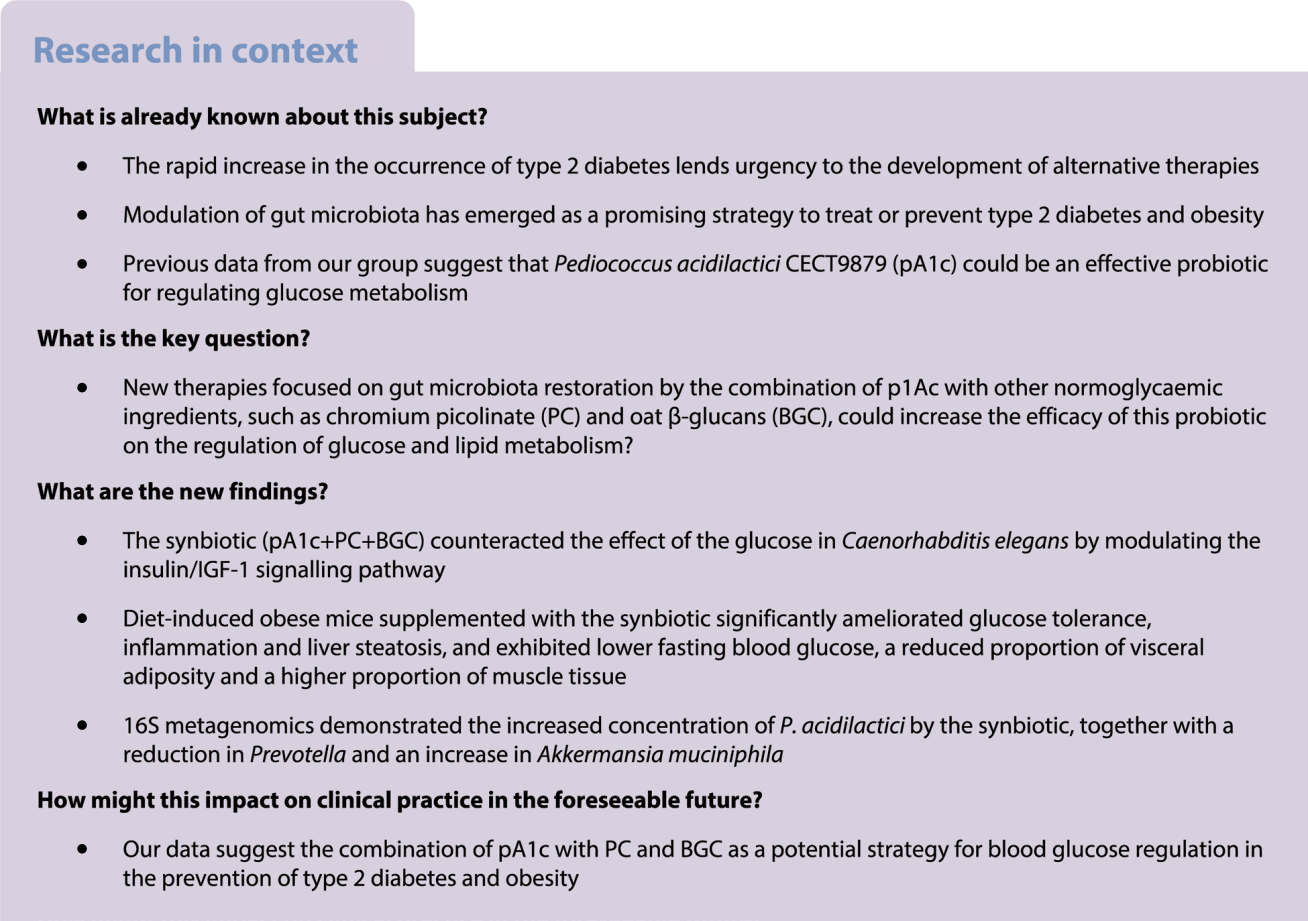


## Introduction

In the recent past, several studies have established a close relationship between diabetes mellitus, insulin resistance, dysbiosis and inflammation [1–5]. One of the most common health complications that accompany diabetes is insulin resistance, which is interconnected with chronic inflammation and dysbiosis, the latter considered to be an imbalance of gut microbiota composition thought to contribute to a range of conditions of ill health [6]. The increasing prevalence of diabetes and its association with the development of other metabolic pathologies requires targeted screening for diabetes and prediabetes in risk groups to identify individuals in the early stages of the disease and treat them as early as possible. Insulin administration and oral glucose-lowering drugs are the main therapy against this disorder. There exist other treatments, such as gene therapy or induced beta cell regeneration [7], although neither these nor previous treatments have been able to curb the high prevalence rate of diabetes. It is therefore crucial to develop novel and alternative therapies to reduce the progression of this pathology and deal with diabetes-related disturbances (CVD [8], retinopathy, vision loss or blindness [9], kidney damage or failure [10], pain and nerve damage [11], dental problems, obesity or chronic inflammation). Thus, it is important to identify compounds with the capacity to normalise blood glucose levels, modulate the intestinal microbiota, reverse the dysbiosis and help to alleviate the problems caused by insulin resistance and diabetes, such as inflammation. With this aim in mind, different probiotics and bioactive compounds have emerged as natural alternatives to the classical treatments of diabetes- and insulin-resistance-related diseases.

Previous studies from our group have demonstrated that the probiotic *Pediococcus acidilactici* CECT9879 (pA1c) counteracts the effect of a high-glucose exposure in *Caenorhabditis elegans* by affecting the insulin signalling pathway (IIS) [12]. Moreover, we observed that the effect of pA1c found in *C. elegans* was also seen in high-fat/high-sucrose (HFS)-induced mice [13, 14]. Thus, we decided to go deeper in the study of the metabolic activities of this probiotic combined with other normoglycaemic compounds and prebiotics. In this work, we first screened and selected the most promising bioactive compounds using *C. elegans* as an experimental model. From among a large number of compounds and combinations, chromium picolinate (PC) and oat β-glucans (BGC) were selected to be mixed with the probiotic. Then, we evaluated the synergistic effects of the combination of pA1c with PC and BGC on the regulation of glucose and lipid metabolism in diet-induced diabetic and obese mice, together with their ability to modulate the gut microbiota composition.

## Methods

### Strains and culture

*Pediococcus acidilactici* strain was deposited, according to the Budapest Treaty, in the Spanish Collection of Type Cultures (CECT), with identification reference CECT 9879, from the proprietary strain collection of Genbioma Aplicaciones (Spain) backed by an international patent ‘Probiotics for regulating blood glucose [PCT/EP2020/087284; WO2021/123355A1]’. This species meets the criteria of qualified presumption of safety (QPS) by the European Food Safety Authority (EFSA). *C. elegans* and pA1c were cultured as described previously [12–16]. The *C. elegans* strains used were: N2 Bristol; *daf-16* (mu86, CF1038) mutant strain; and *daf-16*:GFP mutant strain. All strains were obtained from the Caenorhabditis Genetics Center (CGC; University of Minnesota, MN, USA). *Escherichia coli* OP50 was used as normal nematode diet. All experiments were performed on worms grown and maintained on nematode growth medium (NGM, European bacteriological agar 17.0 g/l, sodium chloride 3.0 g/l, peptone 2.5 g/l, cholesterol 5 mg/l) with or without glucose (10 mmol/l) at 20°C.

### *C. elegans* experimental design

All tests were carried out as described elsewhere [12] in quadruplicate, in six-well cell culture plates with 4 ml NGM or with 4 ml glucose-loaded (10 mmol/l) NGM (NGMg) per well (in the absence or presence of pA1c dispersed throughout the medium). Plates with orlistat (1.5 mg/ml; Sigma Aldrich, St Louis, MO, USA) were used as positive control of fat accumulation reduction. All experiments were performed at a concentration of 5×10^6^ colony-forming units (CFU)/ml of pA1c, 0.5 μg/ml of PC and 50 μg/ml of BGC. After several dose–response experiments to test different concentrations of PC and BGC in *C. elegans* (data not shown), we chose these concentrations as they showed the greatest activity. For all assays, gravid nematodes were subjected to a standard hypochlorite treatment to obtain age-synchronised worms (wild-type or mutants). The eggs were allowed to hatch overnight in M9 medium and about 1000 L1 larvae were transferred onto plates and grown until L4 or 1 day adult stage.

### *C. elegans* assays, image acquisition and quantification

All tests were performed as described elsewhere [12]. Fat accumulation was quantified by Nile Red and Oil Red O (ORO) staining. Fluorescent dye dihydroethidium (DHE; ≥95% purity assessed by HPLA; Sigma Aldrich) was used to quantify levels of reactive oxygen species (ROS) in vivo. Lipofuscin pigment autofluorescence was used as a marker of *C. elegans* ageing. Further, *daf-16*:GFP assay, and egg laying and lifespan analysis were also performed.

For all conditions tested, approximately 300 worms were fixed and stained. Images of Nile Red, ORO and *daf-16*:GFP assays were taken under the same conditions as described elsewhere [12] using a Nikon SMZ18 research stereomicroscope equipped with a Nikon DS-Fi2 high-definition colour camera (Nikon Instruments, Tokyo, Japan). The DHE-labelled ROS formation and the lipofuscin autofluorescence were detected by measuring the fluorescence intensity using a Nikon Eclipse 80i epi-fluorescent microscope, equipped with a TRITC filter (excitation filter 540–625; dichroic mirror 565; barrier absorption 605–655) and DAPI filter (with excitation at 340–380 nm and emission at 435–485 nm), respectively (Nikon Instruments). The image analyses of the Nile Red, ORO, DHE and lipofuscin assays were performed using ImageJ software (http://imagej.nih.gov/ij, 1.53e version, Java 1.8.0_172 [64-bit]). The mean value, calculated as the fluorescence mean value per pixel, together with the integrated density and the volume of the worms, were determined. Approximately 25–40 worms were examined in four independent experiments for each condition.

### C57BL/6J mice experimental design and diets

Ninety-six 4-week-old C57BL/6J male mice (Charles River Laboratories, Saint-Germain-Nuelles, France; RRID:MGI:2159769) were used in this study. The experiment had a total duration of 16 weeks. Mice were kept in an isolated room with controlled temperature (21–23°C) and humidity (50±10%), and a 12 h artificial light–dark cycle. All mice were acclimatised to the experimental facility for 2 weeks, during which time all mice were fed a control diet (2014, Teklad, Global 14% Protein Rodent Maintenance Diet, ENVIGO RMS SPAIN, Barcelona, Spain). Following the acclimation period, mice were randomly divided and allocated into eight groups (*n*=12 mice/group) until the end of the study. Mice were housed four per cage with ad libitum access to water and controlled food intake. Three of the randomly selected cages were maintained on a control diet. To induce diabetes and obesity, seven of the eight groups were fed an HFS diet (D12451; Research Diets, NJ, USA; with 20% of energy corresponding to protein, 35% to carbohydrates and 45% to fat), and fructose (10% wt/vol.) in the water, for 6 weeks. Once HFS-fed groups were stablished, no blinding was carried out. After this period, the fructose was maintained in the water, but the HFS was supplemented with the following treatment for 10 weeks: pA1c group, 1×10^10^ CFU of the probiotic per mouse per day; PC group, 0.1 μg of PC per mouse per day; BGC group, 3.5% of the total daily diet quantity as BGC; pA1c+PC+BGC group, 1×10^10^ CFU of pA1c, 0.1 μg of PC and 3.5% of the total daily diet as BGC per mouse per day; pA1c+PC group, 1×10^10^ CFU of pA1c and 0.1 μg of PC per mouse per day; and pA1c+BGC group, 1×10^10^ CFU of pA1c and 3.5% of the total daily diet as BGC per mouse per day. The HFS control group had no supplement. The remaining group (control-diet group) maintained the control diet (14% of energy as protein) throughout the whole experiment. The PC dose corresponded to a daily intake of 20 μg of PC for an adult of 70 kg (the estimated intake of PC for humans is 25–40 μg/day [17]) and the estimated dose of BGC (3.5%) [18] was based on in vivo models without adverse effects and is equivalent to a dose of 100 g of oat BGC for an adult of 70 kg [19].

The probiotic and synbiotic formulations were prepared every 3 days. The amount of probiotic formulation was kept constant at a daily pA1c dose of 1×10^10^ CFU per mouse. The lactic acid bacteria (LAB) counts in the HFS diet + probiotic formulation were performed by plate counting on MRS agar (Scharlau, Barcelona, Spain) incubated for 48 h at 37°C under CO_2_ atmosphere (5%). Body weight was checked weekly and fasting blood glucose (FBG) by venous tail puncture was measured in weeks 5 and 7 after the supplementation of the different compounds was started. An IPGTT was performed in week 9 of supplementation. All mice were euthanised by decapitation at week 10 of supplementation; trunk blood was collected and serum and plasma samples were obtained for biochemical analysis. Tissue samples from the liver, kidney, spleen, gastrocnemius muscle, white adipose tissue (WAT) depots (mesenteric, retroperitoneal, epididymal and subcutaneous) and brown adipose tissue (BAT) depots were isolated, weighed and immediately stored at −80°C. All procedures were performed by following the national and institutional ethical guidelines of the Care and Use of Laboratory Animals, with the consent of the Food Safety and Environmental Health Service of the Government of Navarra, Spain. The protocol was approved by the Ethics Committee for Animal Experimentation of the University of Navarra (protocol reference 100-21).

### *C. elegans* and C57BL/6J mouse RNA extraction and quantitative real-time PCR analyses

RNA extraction and quantitative real-time PCR (qPCR) analyses were carried out as reported elsewhere [12]. Briefly, Trizol RNA isolation reagent (Thermo Fisher Scientific, Paisley, UK) was used to extract total RNA from *C. elegans* N2 strain and from 100 mg of epididymal fat and gastrocnemius muscle from control, HFS, pA1c, PC, BGC, pA1c+PC, pA1c+BGC, and pA1c+PC+BGC mouse group samples according to the manufacturer’s instructions. A NanoDrop ND-1000 spectrophotometer (Thermo Fisher Scientific, Wilmington, DE, USA) was used to determine the concentration and purity of RNA at 260 nm/280 nm absorption/excitation wavelengths. Afterwards, 1000 ng of RNA was treated with DNAse (AmbionTM DNAse I, RNAse-free; Thermo Fisher Scientific, Waltham, MA, USA) according to the manufacturer’s protocol, and reverse-transcribed into cDNA using 200 U of M-MLV-RT (Invitrogen, Life Technologies, Madrid, Spain) in the presence of 40 U of a recombinant RNAsin RNase inhibitor (Promega, Madison, WI, USA), with an incubation of 10 min at 25°C, 50 min at 37°C and 15 min at 70°C. Gene expression analyses were performed by qPCR using the TaqMan Universal PCR master mix and specific probes from Applied Biosystems Technologies (Thermo Fisher Scientific, Paisley, UK) and Integrated DNA Technologies (Coralville, IA, USA). All reactions were performed using a CFX384 TouchTM Real-Time PCR Detection System (BioRad, Hercules, CA, USA). The expression level of each gene was normalised to the expression of the *pmp-3* gene (used as housekeeping gene control in *C. elegans*), TATA box binding protein (*Tbp*) gene (used as epididymal fat housekeeping gene control in mice) and β-actin (*Actb*) gene (used as gastrocnemius muscle housekeeping gene control in mice) (TaqMan Gene Expression Assays, Life Technologies, Carlsbad, CA, USA). Differences in gene expression between supplemented and non-supplemented worms and mice were quantified using the relative quantification ${2}^{{-\Delta \Delta \mathrm{C}}_{\mathrm{t}}}$ method.

### Blood biochemical variables

In order to determine the lipid profile, serum total cholesterol, HDL-cholesterol (HDL-Chol), triacylglycerol (TG), glucose, aspartate aminotransferase (AST) and alanine aminotransferase (ALT) were quantified with an HK-CP kit (ABX Pentra, Montpellier, France) adapted for a Pentra C200 analyser (HORIBA ABX, Montpellier, France). Serum insulin was quantified with a specific ELISA kit by following the protocol described by the manufacturer (Mercodia, Uppsala, Sweden). Insulin resistance was evaluated by the triglycerides and glucose (TyG) index: Ln ((TG × serum glucose levels (mmol/l))/2). Besides, the log (TG/HDL-Chol) ratio was also calculated to ascertain the atherogenic index of plasma (AIP). Monocyte chemotactic protein-1 (MCP-1) and C-reactive protein (CRP) plasma concentrations were quantified using specific ELISA kits (Thermo Fisher Scientific, Paisley, UK).

### Hepatic TG content

The TG content of the hepatic samples was determined as previously described [20]. Briefly, a 100 mg aliquot of liver from each mouse was dissolved in ethanol with 10% wt/vol. of potassium hydroxide and incubated overnight at 55°C with gentle shaking. The next day, after total tissue digestion, samples were centrifuged for 5 min at 377 *g.* Supernatant fractions were then collected, dissolved in 50% (vol./vol.) ethanol and mixed. After that, samples were mixed 1:1 with magnesium chloride 1 mol/l, incubated for 10 min on ice and then centrifuged for 5 min at 377 *g*. The TG content of these samples was quantified using the HK-CP kit adapted for the Pentra C200 analyser (HORIBA ABX).

### IPGTT

An IPGTT was accomplished during week 9 of supplementation of the study (a week previous to the euthanasia of the mice), as described elsewhere [21]. Mice were fasted for 8 h with free access to water (without fructose) and were then weighed and intraperitoneally administered d-glucose (2 g/kg). Blood glucose was measured before (baseline) and after the glucose administration (at 20, 40, 60, 90 and 150 min) by venous tail puncture using a glucometer and blood glucose test strips (Optium Plus; Abbott Diabetes Care, UK). Glucose content was expressed as mmol/l, and the AUC was calculated according to the formula [21, 22]:

$${\mathrm{AUC}}_{0-180\min}=\lbrack20\times({\mathrm{Glycaemia}}_{\mathrm{baseline}}+2\times{\mathrm{Glycaemia}}_{20\min}+2\times{\mathrm{Glycaemia}}_{40\min}+{\mathrm{Glycaemia}}_{60\min})/2\rbrack+\lbrack30\times({\mathrm{Glycaemia}}_{60\min}+{\mathrm{Glycaemia}}_{90\min})/2\rbrack+\lbrack60\times({\mathrm{Glycaemia}}_{90\min}+{\mathrm{Glycaemia}}_{150\min})/2\rbrack$$

### Faecal sample collection and metagenomic analyses

Faeces from the different groups of mice were collected at week 10 of supplementation and were immediately stored at −80°C in cryotubes for future analyses. DNA isolation and bacterial DNA sequencing analyses were performed at the Cimalab diagnostics genomics Unit of the Center for Applied Medical Research (Pamplona, Spain). dsDNA characterisation was performed with Qubit (Thermo Fisher Scientific, Paisley, UK). Regions V3 and V4 of the ribosomal 16s gene were sequenced by using Illumina protocols described elsewhere [23]. Briefly, sequencing consisted of two PCR reactions in which the V3 and V4 regions of the 16S rRNA gene were amplified. This required the use of 16S-forward and 16S-reverse specific primers (16S amplicon PCR forward primer = 5′TCGTCGGCAGCGTCAGATGTGTA TAAGAGACAGCCTACGGGNGGCWGCAG; 16S amplicon PCR reverse primer = 5′GTCTCGTGGGCTCGGAGATGTGTATAAGAGACAGGACTACHVGGGTATCTAATCC; Nextera XT DNA Index Kit FC-131–1002; Illumina; San Diego, CA, USA). The protocol followed for the first PCR was at 95°C for 3 min and 25 cycles of 95°C for 30 s, 55°C for 30 s, 72°C for 30 s and 72°C for 5 min. After the cleansing process, 5 μl were taken from the first PCR sample to use for the second PCR. For the second PCR, the protocol followed was 95°C for 3 min and eight cycles of 95°C for 30 s, 55°C for 30 s, 72°C for 30 s and 72°C for 5 min. A cleansing process was performed after each PCR to remove the primers from the sample. Afterwards, the samples were loaded into the MiSeq equipment for sequencing and quantification. The operational taxonomic units (OTUs) grouping methods were used to analyse the gut microbiome. Taxonomy was assigned using BLAST (https://blast.ncbi.nlm.nih.gov/Blast.cgi) and HITdb (v1.00, http://www.hitdb.com). The alignments of the different OTUs (sequences) were conducted with the workflow of 16S Metagenomics of Illumina database, which allows the classification at the level of phylum, class, order, family, genus or species. The abundance matrices were filtered and then normalised at each level of classification. Bioinformatic analysis of the differences in the gut microbiota of the different groups was performed using ALDEx2 R package (https://www.bioconductor.org/packages/release/bioc/html/ALDEx2.html; bioconductor version: Release 3.17).

### Statistical analyses

All animals of the initial study were included in the analysis, without exclusion. *C. elegans* body fat (Nile Red and ORO), together with oxidative stress (DHE), lipofuscin determinations, real-time PCR data (gene expression) and C57BL/6J mouse physiological, tissue and biochemical variables, gene expression, and AUC of the IPGTT were evaluated by the one-way ANOVA test followed by the Student–Newman–Keuls (SNK) multiple comparisons test when statistical significance (*p*<0.05) was reached in the ANOVA test. For lifespan assays, logrank (Mantel–Cox test) between synbiotics and control treatments were performed. All tests were carried out using StataSE v14 software (StataCorp, College Station, TX, USA).

## Results

### **The synbiotics improve *C. elegans ***health in comparison with the control and the pA1c alone

**Fig. 1:**
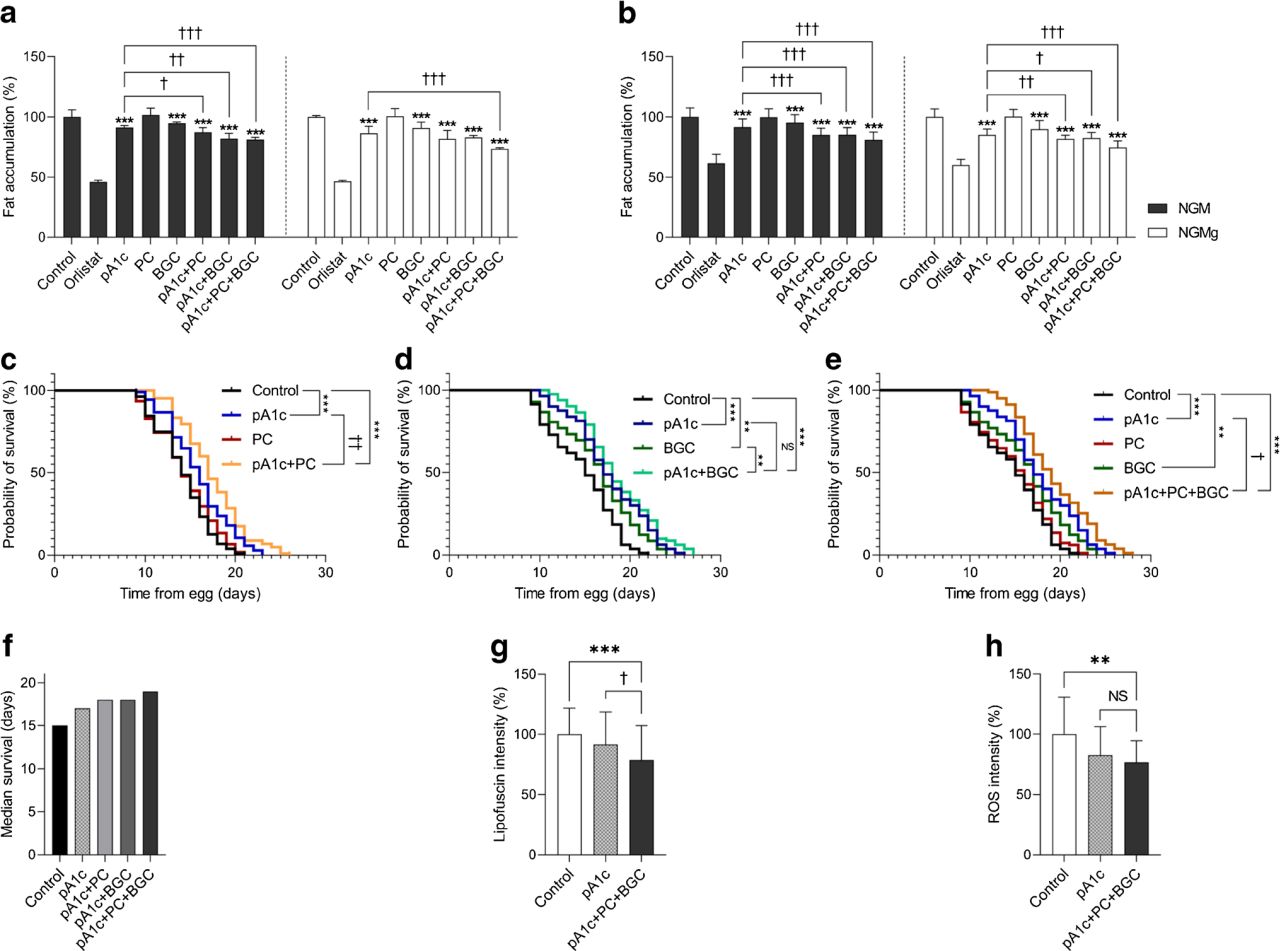
The synbiotics reduce fat accumulation in *C. elegans* and increase the worm’s lifespan in comparison with the control and pA1c alone. (**a**) Nile Red staining quantification of control and supplemented worms in NGM and in NGMg. (**b**) ORO staining quantification of control and supplemented worms in NGM and in NGMg. Results are expressed as the mean ± SD relative to control worms. ***p<0.001 vs control worms; ^†^*p*<0.05, ^††^*p*<0.01 and ^†††^*p*<0.001 vs pA1c (one-way ANOVA test followed by the SNK multiple comparisons test when statistical significance [*p*<0.05] was reached in the ANOVA test). (**c**–**f**) Lifespan analyses (**c**–**e**) and median survival time (**f**) of the different supplemented groups in NGM. **p<0.01 and ***p<0.001 vs water-treated control worms; ^NS^*p*>0.05, ^†^*p*<0.05 and ^††^*p*<0.01 vs pA1c-supplemented worms (logrank Mantel–Cox test). (**g**, **h**) Lipofuscin ageing pigment (**g**) and ROS production (**h**) quantification of water-, pA1c- and pA1c+PC+BGC-treated worms in NGMg medium

Previous work by our group revealed that supplementation with pA1c reduces fat accumulation and lipid content in *C. elegans* [12]. Thus, we first screened different synbiotics containing pA1c, modulating *C. elegans* lipid homeostasis, by ascertaining their reducing effect on lipid accumulation through Nile Red and ORO staining methods [12, 24]. Water-treated worms were used as control, and orlistat-treated worms were used as positive control [25]. All synbiotic combinations containing pA1c (pA1c+PC, pA1c+BGC and pA1c+PC+BGC) reduced lipid accumulation in wild-type *C. elegans* grown in NGM (by 12.7%, 17.9% and 18.7%, respectively), and were even more effective in *C. elegans* grown in NGMg (NGM loaded with glucose 10 mmol/l) (18.2%, 17% and 26.5%, respectively), with respect to the corresponding control worms, quantified by Nile Red staining (Fig. 1a). The results obtained with Nile Red were confirmed by ORO staining (Fig. 1b). Besides, it was found that the three synbiotics combined significantly reduced nematode fat accumulation when compared with the probiotic alone.

Then, we evaluated the synergistic activity between pA1c, PC and BGC over other variables of nematode health. In a previous study, we demonstrated that pA1c improved ageing and increased lifespan in *C. elegans* [12]. Here, we verified the effectiveness of pA1c alone in increasing the lifespan when compared with water treatment of worms; we also observed that the nematodes supplemented with the three synbiotics lived longer than the worms supplemented with only pA1c (Fig. 1c–e). This increase in worm lifespan was statistically significant for pA1c+PC (Fig. 1c) and pA1c+PC+BGC supplementation (Fig. 1e) when compared with pA1c supplementation. In fact, we observed that pA1c+PC and pA1c+BGC supplementation resulted in higher values of median survival time (18 days) than pA1c alone (17 days), and this effect was stronger with the triple combination pA1c+PC+BGC (19 days) (Fig. 1f).

The fat-reduction activity and the extension of the lifespan observed with the synbiotic pA1c+PC+BGC led us to evaluate its effectiveness in metabolic pathways related to the health of the worm (oxidative stress, development and ageing). Supplementation with pA1c+PC+BGC, from larval stage 1 to larval stage 4 in NGMg plates, significantly reduced the autofluorescence of lipofuscin (78.7% of lipofuscin), both when compared with water-treated control worms (100% of lipofuscin) and when compared with pA1c alone (91.6% of lipofuscin) (Fig. 1g). Moreover, the synbiotic reduced the levels of ROS induced by the glucose (by 23.3% compared with water-treated control worms) (Fig. 1h). Furthermore, pA1c+PC+BGC exhibited lower values of ROS (76.7%) than pA1c (82.5%), demonstrating once again the existence of a synergistic effect between pA1c, PC and BGC (Fig. 1h).

Finally, no differences were observed in the time of appearance of both eggs and L1 larvae when comparing pA1c+PC+BGC- and water-supplemented plates 72 h after hatching, in either NGM (electronic supplementary material [ESM] Fig. 1a) or NGMg (ESM Fig. 1b). This result suggests that supplementation with the synbiotic pA1c+PC+BGC did not affect the correct development of *C. elegans*.

### The synbiotics modulate the IIS and the fatty acid metabolic pathway in ***C. elegans***

**Fig. 2:**
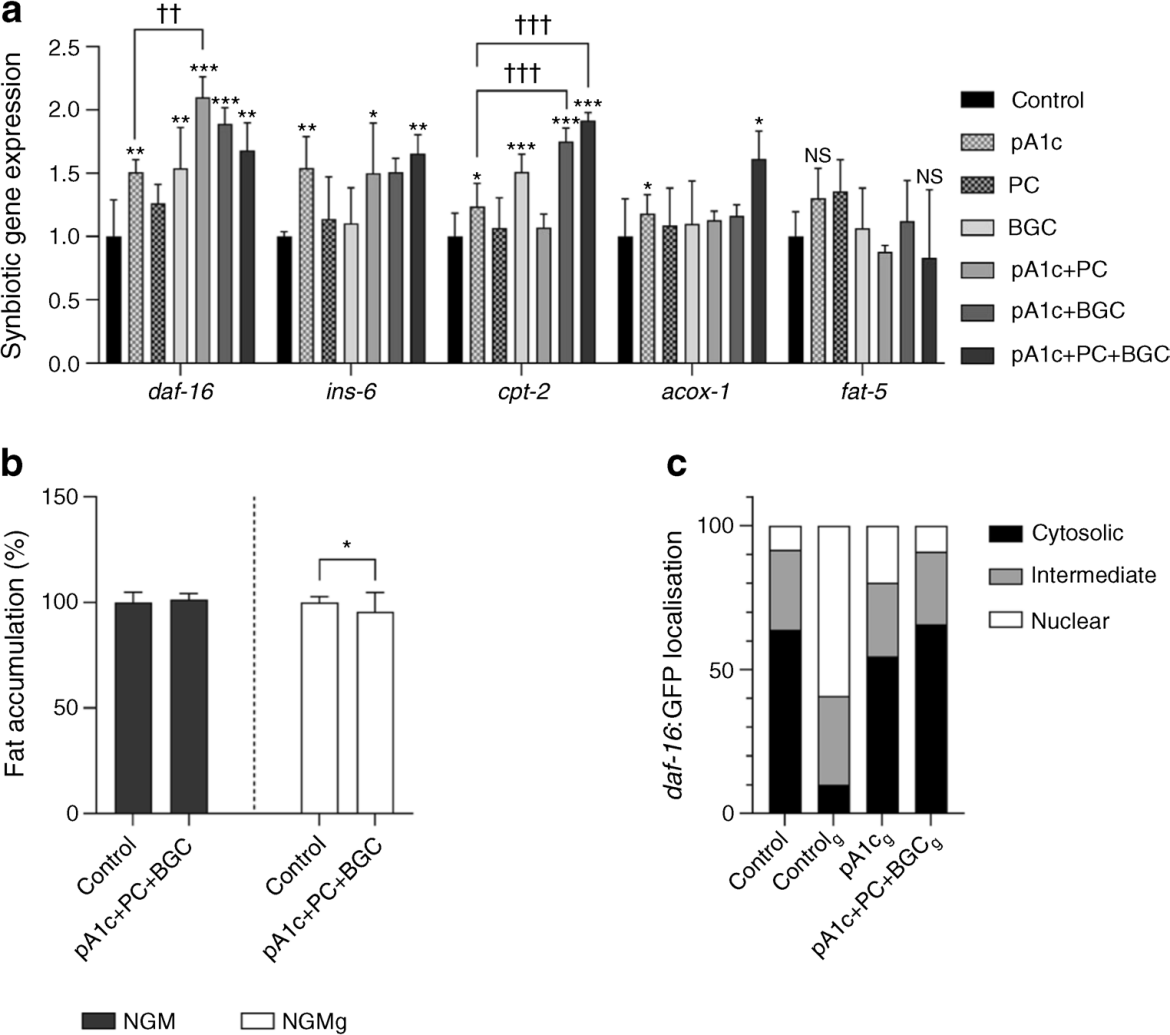
(**a**) Gene expression analyses, quantified by qPCR, in *C. elegans* following supplementation with pA1c+PC, pA1c+BGC, and pA1c+PC+BGC. Results are expressed as the mean ± SD relative to control worms (*n*=8 per group). Gene expression levels were normalised to the housekeeping gene *pmp-3*. Data were generated using the ${2}^{{-\Delta \Delta \mathrm{C}}_{\mathrm{t}}}$ method. ^NS^*p*>0.05, *p<0.05, **p<0.01 and ***p<0.001 vs control worms; ^††^*p*<0.01 and ^†††^*p*<0.001 for synbiotics vs other supplemented groups (one-way ANOVA test followed by the SNK multiple comparisons test when statistical significance [*p*<0.05] was reached in the ANOVA test [SNK multiple comparison test]). (**b**) Nile Red staining quantification of fat accumulation in water-treated control worms and pA1c+PC+BGC-supplemented worms in NGM or NGMg in a *daf-16* mutant of *C. elegans*. *p<0.05 (Student’s *t* test). (**c**) Classification of worms according to *daf-16*:GFP localisation

Our gene expression analyses (Fig. 2a) demonstrated that the three synbiotics increased the expression of *daf-16* (the main *C. elegans* gene involved in glucose metabolism) in comparison with pA1c alone. The increase was only statistically significant for the pA1c+PC combination; this combination did not result in any difference in the expression of other genes related to fatty acid β-oxidation or biosynthesis. The combination pA1c+BGC, apart from the upregulation of *daf-*16, resulted in a significant upregulation in *cpt-2*, which is involved in fatty acid β-oxidation, when compared with pA1c alone. Furthermore, supplementation with the triple combination pA1c+PA+BGC resulted in an upregulation in *daf-16*, *ins-6*, *cpt-2* and *acox-1* (the latter being one of the main genes involved in fatty acid β-oxidation), with a significant increase in *cpt-2* gene expression when compared with pA1c alone. These results demonstrate that, as in the fat accumulation and lifespan assays, the triple combination of pA1c+PA+BGC exhibits a synergistic activity over pA1c alone, and these effects are mediated by the IIS and the fatty acid degradation (β-oxidation) pathway.

To shed light on the mechanism of action of the triple synbiotic, and to analyse in more detail its involvement in IIS, *daf-16* and *daf-16*:GFP mutants were used. Previous studies established that the *daf-16* mutation overturns the pA1c-mediated body-fat-reducing effect and that the fat-reducing effect of pA1c alone requires not only *daf-16* activity but also the IIS [12]. In this work, pA1c+PC+BGC did not reduce the fat accumulation in *daf-16* mutant worms in NGM (Fig. 2b). When glucose was added to the medium, the synbiotic was able to reduce the lipid droplets by 5% when quantified by Nile Red, in comparison with the control worms (Fig. 2b).

Finally, our previous studies have reported that pA1c inhibits the high-glucose-induced nuclear translocation of *daf-16* by affecting the IIS. In normal conditions, *daf-16* remains in the cytosol of the cells of the worm but when glucose is added to the medium *daf-16* translocates to the nucleus of the cells of *C. elegans* as a defensive action from the worm to cope with the stress induced by glucose. In this study, we observed that in pA1c+PC+BGC-supplemented worms the glucose nuclear localisation of *daf-16* was reversed in a more effective way than in worms supplemented with pA1c alone (Fig. 2c).

### The synbiotics combinations including pA1c reduce FBG and significantly ameliorate glucose tolerance after an IPGTT in mice

Previous studies have established the relationship between reduction and maintenance of blood glucose levels, and pA1c [13], PC [26, 27] and BGC [28], indicating the presumed potential activity of the probiotic and the two bioactive compounds in the regulation of glucose metabolism and insulin sensitivity. Therefore, we evaluated the synergic effect of our synbiotic combinations on mammalian glucose homeostasis and insulin sensitivity in C57BL/6J mice with diabetes and diet-induced obesity.

**Fig. 3:**
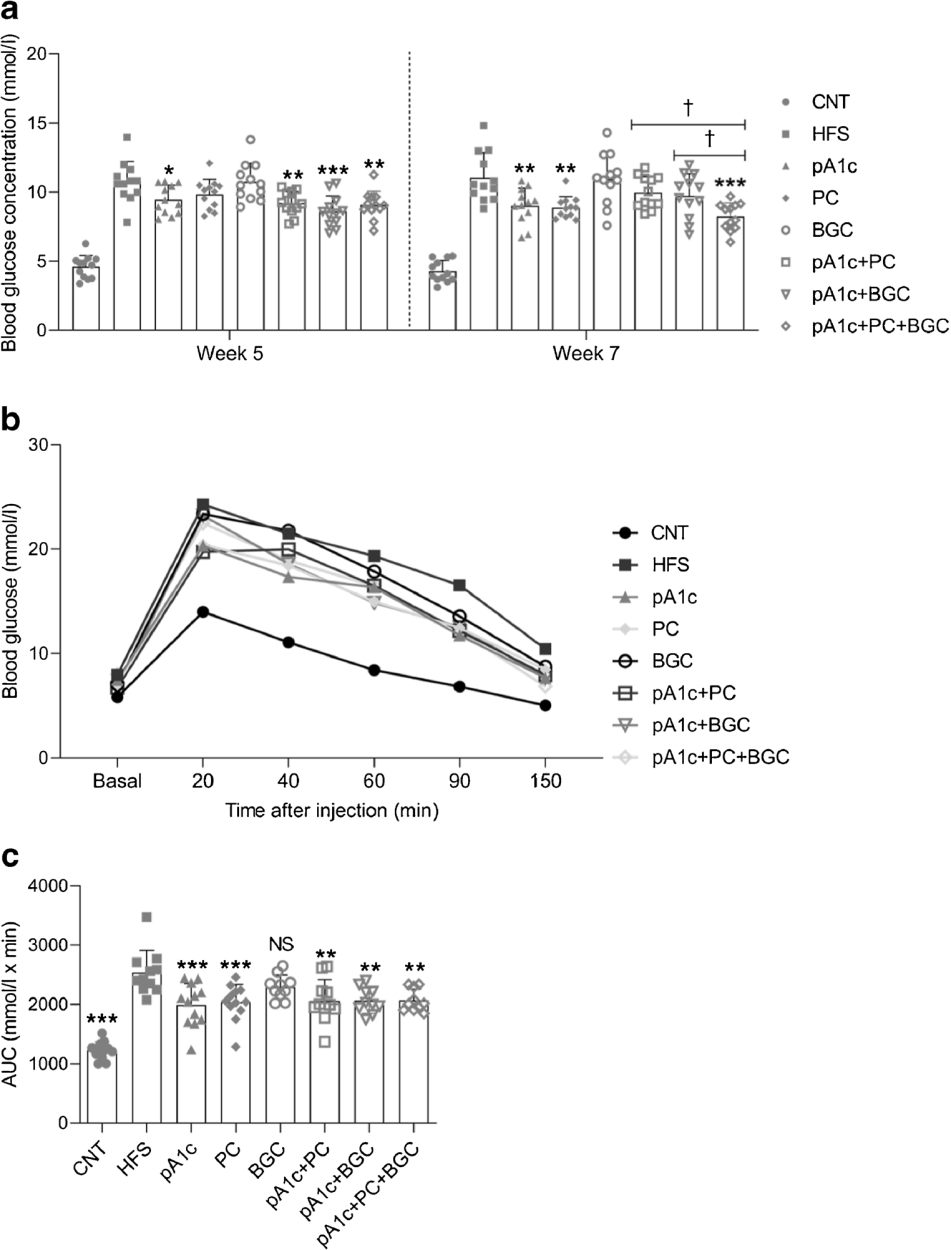
FBG monitoring in C57BL/6J mice supplemented with synbiotics. (**a**) Effects of synbiotics on FBG during week 5 and week 7 of supplementation. (**b**, **c**) Effects of synbiotics on blood glucose levels after the IPGTT performed during week 9 of supplementation; the total AUC is shown (**c**). Results are expressed as the mean ± SD (*n*=12 per group). ^NS^*p*>0.05, *p<0.05, **p<0.01 and ***p<0.001 vs HFS group; ^†^*p*<0.05 for triple synbiotic vs the other synbiotic-supplemented groups (one-way ANOVA test followed by the SNK multiple comparisons test when statistical significance [*p*<0.05] was reached in the ANOVA test [SNK multiple comparison test]). CNT, control

During this study, we monitored blood glucose levels throughout the whole experiment. Blood glucose levels were measured by venous tail puncture at weeks 5 and 7 of supplementation and we carried out an IPGTT at week 9 from the beginning of the supplementation (Fig. 3). At week 5 of the supplementation, FBG was significantly reduced in all the pA1c-supplemented groups compared with the HFS group (Fig. 3a). What is more, the second FBG quantification at week 7 reflected similar data. In accordance with the *C. elegans* results, pA1c+PC+BGC-supplemented mice exhibited lower FBG values in week 7 than in week 5 of supplementation (Fig. 3a.), suggesting that this triple synbiotic is capable of maintaining FBG, and reducing it throughout the supplementation. Furthermore, at week 7, significant differences between pA1c+PC+BGC and the other two synbiotics (pA1c+PC and pA1c+BGC) were observed (Fig. 3a), confirming the effectiveness of the triple combination.

One week prior to euthanasia of the mice (week 9 of supplementation), an IPGTT was performed. As in the case of FBG at week 5, all mice belonging to the groups supplemented with the probiotic (pA1c, pA1c+PC, pA1c+BGC, pA1c+PC+BGC), together with the PC group, experienced a significantly lower increase in glucose levels at 40, 60 and 90 min after the glucose load, when compared with mice in the non-supplemented HFS group (Fig. 3b). The glucose levels in the non-supplemented HFS group remained higher at 150 min after the glucose load, compared with all supplemented groups (Fig. 3b). Quantification of the total AUC demonstrated that all groups with the probiotic in their supplementation (pA1c, pA1c+PC, pA1c+BGC, pA1c+PC+BGC), together with the PC group, exhibited an AUC significantly smaller than that of the HFS group (Fig. 3c). No significant differences were observed among the supplemented groups. Overall, the IPGTT analysis demonstrated that the supplementation with pA1c, PC, pA1c+PC, pA1c+BGC and pA1c+PC+BGC attenuated the postprandial hyperglycaemia observed after the i.p. glucose load and contributed to reducing and normalising blood glucose levels in mice.

### The synbiotic combination of pA1c, PC and BGC improves liver markers and reduces inflammation in mice

After euthanasia of mice, we analysed glucose metabolism to clarify the mechanism of action of these synbiotics. As shown in Table 1, there were no differences in fasting glucose levels between the HFS group and the supplemented groups, except for the pA1c group, which presented significantly lower values of glucose than the HFS mice. Regarding serum insulin levels, all the groups supplemented with the probiotic (pA1c, pA1c+PC, pA1c+BGC and pA1c+PC+BGC) exhibited lower values than the HFS group, with no significant differences between them and the control group.

**Table 1:** Biochemical variables in C57BL/6J mice under normal and experimental conditions

Variable	Control	HFS	pA1c	PC	BGC	pA1c+PC	pA1c+BGC	pA1c+PC+BGC	ANOVA (*p* value)
Glucose metabolism biomarkers									
Glucose (mmol/l)	6.86±0.2^a^	12.75±0.3^b^	10.69±0.4^c^	12.07±0.2^b,c^	11.98±0.4^b,^^**c**^	11.58±0.7^b,c^	11.31±0.4^b,c^	11.38±0.2^b,c^	<0.001
Insulin (pmol/l)	72.1±4.4^a^	240.3±41.0^b^	172.8±29.5^a,b^	241.9±36.9^b^	231.5±29.6^b^	126.0±14.4^a,b^	173.2±21.0^a,b^	126.7±10.6^a,b^	<0.001
Cholesterol metabolism biomarkers									
Chol (mmol/l)	2.77±0.07^a^	4.96±0.15^b^	4.58±0.29^b^	4.97±0.14^b^	4.90±0.19^b^	4.58±0.12^b^	4.73±0.12^b^	4.48±0.13^b^	<0.001
HDL-chol (mmol/l)	1.46±0.04^a^	1.90±0.05^b^	1.86±0.10^b^	1.90±0.04^b^	1.81±0.05^b^	1.93±0.03^b^	1.84±0.02^b^	1.83±0.04^b^	<0.001
Total cholesterol/HDL-chol	1.90±0.04^a^	2.61±0.07^b^	2.46±0.05^b^	2.62±0.06^b^	2.70±0.08^b^	2.37±0.04^b^	2.57±0.06^b^	2.45±0.04^b^	<0.001
TG (mmol/l)	0.94±0.03^a^	1.28±0.05^b^	1.13±0.11^a,b^	1.29±0.08^b^	1.16±0.05^a,b^	1.11±0.08^a,b^	1.15±0.07^a,b^	1.14±0.07^a,b^	<0.05
TyG index^d^	4.61±0.02^a^	5.05±0.02^b^	4.85±0.08^b^	5.05±0.04^b^	4.99±0.02^b^	4.91±0.05^b^	4.95±0.02^b^	4.94±0.06^b^	<0.001
AIP index^e^	0.16±0.02	0.19±0.02	0.13±0.03	0.18±0.03	0.17±0.02	0.10±0.04	0.13±0.02	0.14±0.03	NS
Hepatic metabolism biomarkers									
ALT (µkat/l)	0.88±0.03^a^	1.22±0.16^b^	1.28±0.08^b^	1.44±0.12^b^	1.07±0.06^a,b^	1.12±0.07^a,b^	0.91±0.04^a^	0.98±0.06^a^	<0.001
AST (µkat/l)	5.73±0.61^a^	8.47±0.74^b^	6.60±0.45^a^	6.86±0.30^a^	6.34±0.54^a^	6.06±0.36^a^	5.84±0.46^a^	6.08±0.35^a^	<0.05

Data are expressed as mean ± SEM

^a^Statistically equal to the control group

^b^Statistically equal to the HFS group

^c^Statistically different from the control and HFS groups

^d^TyG index was calculated with the formula: TyG = log_*e*_ (TG [mmol/l] × glucose [mmol/l]/2)

^e^AIP index was calculated with the formula: AIP = log_10_ (TG/HDL-chol)

Statistical analyses were performed using the one-way ANOVA test followed by the SNK multiple comparisons test when statistical significance (*p*<0.05) was reached in the ANOVA test between the eight groups

**Fig. 4:**
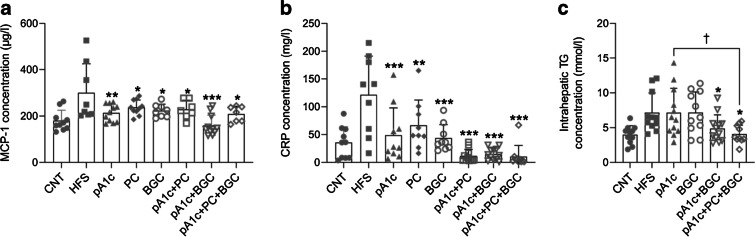
(**a**) MCP-1 levels in control and supplemented C57BL/6J mice, quantified by ELISA. (**b**) CRP levels in control and supplemented mice quantified by ELISA. (**c**) Intrahepatic TG levels in control and supplemented mice. Results are expressed as the mean ± SD (*n*=12 per group). *p<0.05, **p<0.01 and ***p<0.001 vs HFS group; ^†^*p*<0.05 vs pA1c group (one-way ANOVA test followed by the SNK multiple comparisons test when statistical significance [*p*<0.05] was reached in the ANOVA test [SNK multiple comparison test]). CNT, control

Delving deeper into inflammation and liver damage, intrahepatic TGs were quantified and, in addition, the levels of two different inflammatory markers were measured: MCP-1; and CRP. A decrease in the circulating levels of these two proinflammatory biomarkers was observed in all supplemented groups of mice, when compared with the high-fat group (Fig. 4a,b). Besides, almost all of the groups supplemented with pA1c exhibited even lower levels of these two key inflammatory proteins than those in control mice (especially CRP, where combination of the three synbiotics reduced the concentration of this protein compared with the probiotic alone, showing a synergistic effect), suggesting an anti-inflammatory activity of the synbiotic. Furthermore, a downregulation of intrahepatic TG content was found in mice supplemented with pA1c+BGC and pA1c+PC+BGC when compared with the HFS group (Fig. 4c). What is more, the triple combination showed TG levels even lower than those in the control group and TG levels were significantly reduced when compared with the pA1c group, demonstrating once again the synergistic effect of this synbiotic. Altogether, we can conclude that supplementation with the synbiotics, especially pA1c+PC+BGC and pA1c+BGC, produced a synergistic effect that reduced liver damage and inflammation, and normalised hepatic biomarkers.

### The synbiotic combination of pA1c, PC and BGC counteracts the effect of the diet over adipose tissue accumulation in mice

No differences were observed in the body weight of the HFS-fed mice after the different supplementations, nor in the weight of the liver, spleen and kidney (Table 2). However, mice supplemented with the triple synbiotic pA1c+PC+BGC presented significantly higher weight of the gastrocnemius muscle. Additionally, mice supplemented with pA1c+PC+BGC exhibited significantly reduced mesenteric and epididymal fats compared with the HFS group. Moreover, supplementation with the triple combination pA1c+PC+BGC significantly reduced the visceral WAT fat (mesenteric fat + epididymal fat + retroperitoneal fat) and the total WAT (visceral WAT + subcutaneous fat) when compared with HFS and pA1c supplementation alone, suggesting a synergistic effect between the probiotic and the bioactive compounds similar to that seen in the *C. elegans* experiments. Furthermore, the pA1c+PC+BGC triple combination significantly increased the BAT weight when compared with the HFS group. These results demonstrate that supplementation with pA1c+PC+BGC alleviates metabolic syndrome-related disturbances.

**Table 2:** Body weight and tissue weights in C57BL/6J mice under normal and experimental conditions

Tissue	Control	HFS	pA1c	PC	BGC	pA1c+PC	pA1c+BGC	pA1c+PC+BGC	ANOVA (*p* value)
Body weight (BW) (g)	25.47±0.78^a^	37.26±1.18^b^	37.63±1.12^b^	38.82±1.23^b^	38.52±1.07^b^	36.75±1.34^b^	38.18±1.40^b^	38.69±1.19^b^	<0.001
Tissue weight (prop. total BW)									
Liver	1.05±0.10	1.12±0.05	1.18±0.05	1.21±0.05	1.23±0.09	1.18±0.09	1.13±0.06	1.17±0.05	NS
Spleen	0.07±0.01	0.09±0.01	0.08±0.01	0.09±0.01	0.08±0.01	0.08±0.01	0.08±0.01	0.09±0.01	NS
Kidney	0.17±0.01	0.18±0.01	0.18±0.01	0.18±0.01	0.18±0.01	0.18±0.01	0.18±0.01	0.18±0.01	NS
Gastrocnemius muscle	0.15±0.01^a^	0.16±0.01^a^	0.17±0.01^a^	0.17±0.01^a^	0.17±0.0^a^	0.17±0.01^a^	0.17±0.01^a^	0.19±0.01^b^	<0.01
WAT depot weight (prop. total BW)									
Mesenteric fat	0.54±0.09^a^	2.05±0.13^**b**^	1.41±0.17^c^	1.80±0.16^b^	1.67±0.15^b^	1.19±0.17^c^	1.58±0.13^b^	1.33±0.12^c^	<0.001
Epididymal fat	1.74±0.29^a^	6.05±0.17^**b**^	5.17±0.53^b^	5.37±0.25^b^	5.48±0.30^b^	5.14±0.29^b^	5.21±0.31^b^	4.36±0.31^c^	<0.001
Subcutaneous fat	0.41±0.08^a^	1.90±0.22^b^	1.58±0.25^b^	1.81±0.19^b^	1.82±0.15^b^	1.71±0.22^b^	1.64±0.24^b^	1.39±0.10^b^	<0.001
Retroperitoneal fat	0.35±0.07^a^	1.43±0.10^b^	1.32±0.15^b^	1.53±0.10^b^	1.57±0.12^b^	1.57±0.13^b^	1.42±0.12^b^	1.25±0.11^b^	<0.001
Visceral WAT^d^	2.63±0.34^a^	9.07±0.32^b^	7.90±0.58^b^	8.70±0.30^b^	8.71±0.41^b^	7.90±0.48^b^	8.21±0.38^b^	7.23±0.37^c^	<0.001
Total WAT^e^	3.04±0.41^a^	10.97±0.43^b^	9.49±0.79^b^	10.51±0.39^b^	10.53±0.47^b^	9.61±0.57^b^	9.85±0.52^b^	8.51±0.39^c^	<0.001
BAT depot weight (prop. total BW)									
Brown fat	0.25±0.02^a^	0.40±0.01^b^	0.44±0.07^b^	0.46±0.03^b^	0.52±0.03^b^	0.47±0.03^b^	0.50±0.04^b^	0.58±0.06^c^	<0.001

Data are expressed as mean ± SEM

^a^Statistically equal to the control group

^b^Statistically equal to the HFS group

^c^Statistically different from the control and HFS groups

^d^Visceral WAT, sum of mesenteric, epididymal and retroperitoneal fat depots

^e^Total WAT, sum of visceral WAT and subcutaneous fat

Statistical analyses were performed using the one-way ANOVA test followed by the SNK multiple comparisons test when statistical significance (*p*<0.05) was reached in the ANOVA test between the eight groups

prop., proportion

### The synbiotic combinations including pA1c have blood glucose-regulating effects in mice and alleviate insulin resistance by affecting insulin signalling and promoting β-oxidation

**Fig. 5:**
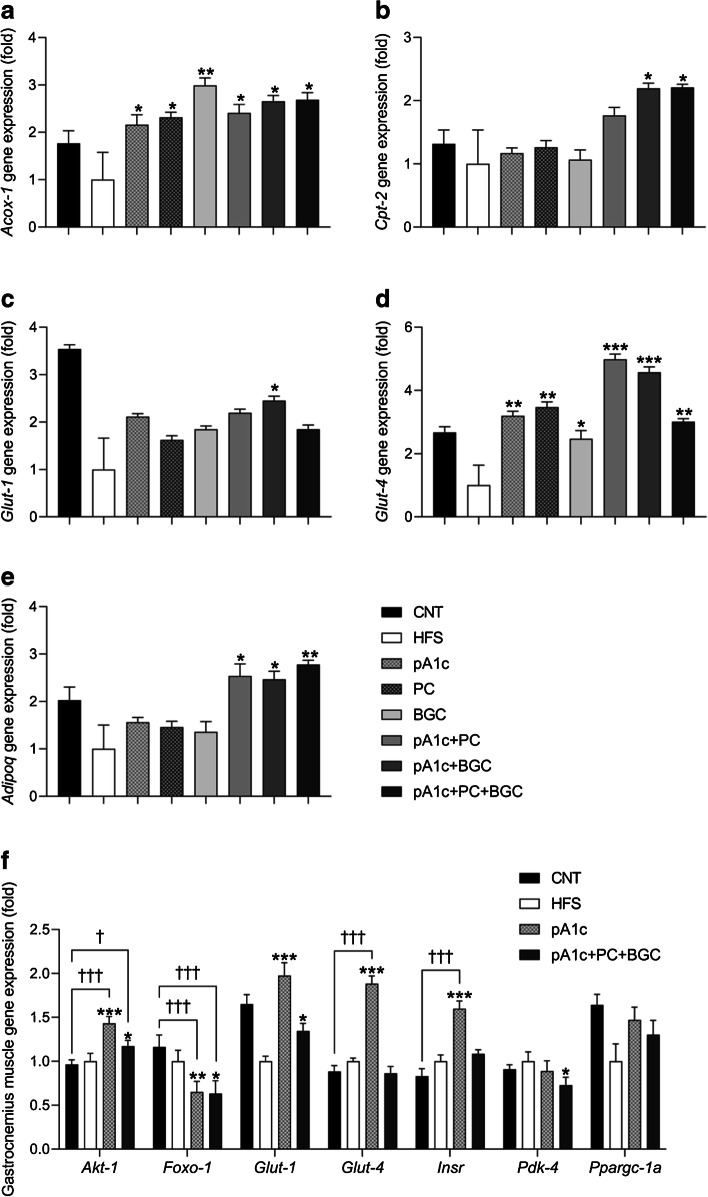
Epididymal fat (**a**–**e**) and gastrocnemius muscle (**f**) gene expression analysis of key genes involved in glucose metabolism and the biosynthesis and degradation of lipid metabolism, quantified by qPCR in mice. Results are expressed as the mean ± SD relative to HFS mice (*n*=12 per group). Epididymal fat gene expression levels were normalised to the housekeeping gene *Tbp* and gastrocnemius muscle gene expression levels were normalised to the housekeeping gene *Actb*. Data are expressed using the ${2}^{{-\Delta \Delta \mathrm{C}}_{\mathrm{t}}}$ method. *p<0.05, **p<0.01 and ***p<0.001 vs HFS group; ^†^*p*<0.05 and ^†††^*p*<0.001 vs control group (one-way ANOVA test followed by the SNK multiple comparisons test when statistical significance [*p*<0.05] was reached in the ANOVA test [SNK multiple comparison test]). CNT, control

Gene expression analyses in epididymal fat (Fig. 5a–e) showed an upregulation of *Glut-1* and *Glut-4* (key genes of the glucose metabolism, also known as *Slc2a1* and *Slc2a4*, respectively) in the groups supplemented with the synbiotic, compared with the HFS group. However, no synergistic effect compared with pA1c alone was observed. Moreover, it was also shown that supplementation with the synbiotics including pA1c produced an overexpression of *Acox-1* and *Cpt-2* (also known as *Acox1* and *Cpt2*, respectively), which are fundamental β-oxidation genes. These results are in accordance with *C. elegans* data, demonstrating that the mechanism of action of the synbiotic affected insulin signalling and fatty acid β-oxidation. Importantly, supplementation with the synbiotic increased the expression of *Adipoq*, encoding adiponectin (Fig. 5e), a cytokine secreted by the adipose tissue that regulates energy metabolism, stimulates fatty acid oxidation, reduces plasma TG and improves glucose metabolism by increasing insulin sensitivity. Thus, its upregulation, together with the increase in β-oxidation activity, the modulation of the IIS and the reduction of plasma TG shed light on the mechanism by which the synbiotics improves glucose and insulin metabolism.

To confirm the role of pA1c in glucose signalling, we also investigated the gene expression of different key IIS genes in gastrocnemius muscle (Fig. 5f). These analyses confirmed *Glut-1* and *Glut-4* overexpression in skeletal muscular tissue compared with the HFS group. Further, an increased expression of *Akt-1* (also known as *Akt1*) and *Insr* in mice that had received the probiotic was observed in comparison with HFS mice. Furthermore, pA1c-supplemented mice presented even higher levels than control mice. On the other hand, probiotic- and synbiotic-supplemented mice presented significantly decreased expression of *Foxo-1* and *Pdk-4* (also known as *Foxo1* and *Pdk4*, respectively) compared with the HFS group.

### The synbiotics showed beneficial effects on the faecal microbiota composition of mice

ESM Table 1 summarises the statistically significant microbial changes in C57BL/6J mice following supplementation with pA1c, PC or BGC. It is important to mention that each section of ESM Table 1 includes all the groups that received that supplement, and all are compared with the HFS group: the pA1c section includes all the groups that were supplemented with pA1c (pA1c group, pA1c+PC, pA1c+BGC and pA1c+PC+BGC); the PC section includes the PC group, pA1c+PC group and pA1c+PC+BGC group; and the BGC section included the BGC group, pA1c+BGC group and pA1c+PC+BGC group. Families and genera listed in each section are common to all the groups that were supplemented with that compound, and are restricted to only one section, which means that the same family or genera can only appear in one section (the supplemented group with the highest abundance of the microorganism). Focusing on the pA1c section of ESM Table 1, we can observe an increase in the abundance of the Oceanospirillaceae and Saprospiraceae families, and a decrease in the abundance of genera such as *Asaccharobacter* (this bacterium has been found to be increased in people with depression [29]) and *Saccharofermentans* (16S rDNA sequencing in other studies showed that increasing dietary energy significantly stimulated the relative abundance of *Saccharofermentans* [30]) in comparison with the HFS group.

ESM Table 1 highlights differences in the abundance of very important families and genera of the intestinal microbiota following supplementation of mice with PC and BGC. There was a decrease in the abundance of the families Eubacteriaceae, Christensenellaceae, Lachnospiraceae and Ruminococcaceae and an increase in Bifidobacteriaceae, Lactobacillaceae and Verrucomicrobiaceae. Differences in the abundance of genera following PC and BGC supplementation included a decrease in *Alloprevotella*, *Christensenella*, *Lachnospira*, *Odoribacter* and *Ruminococcus* among others, and an increase in *Akkermansia*, *Bifidobacterium* and *Lactobacillus*. All families (ESM Table 2) and genera (ESM Tables 3, 4 and 5) that were significantly changed following BGC supplementation are listed in the ESM.

**Fig. 6:**
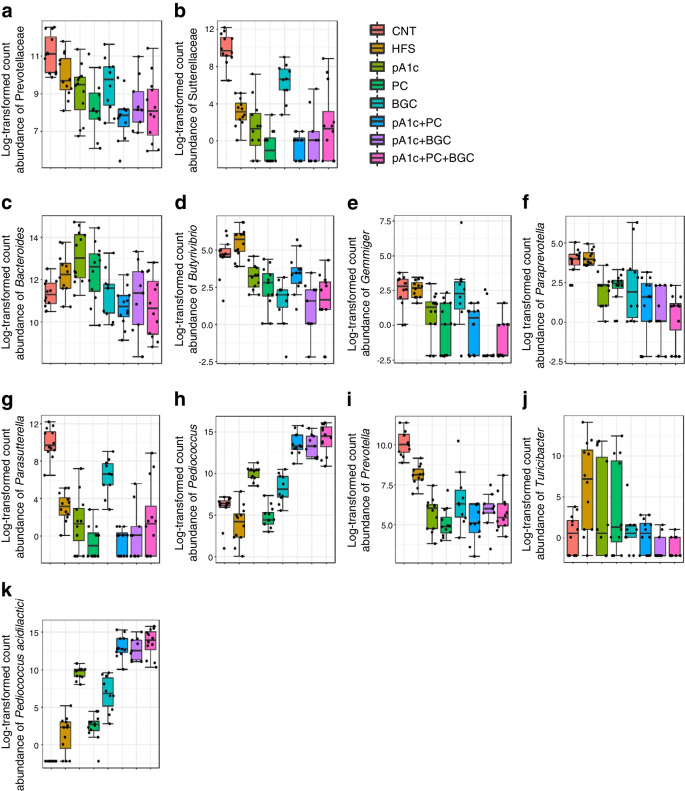
Bacterial families (**a**, **b**), genera (**c**–**j**) and species (**k**) differential between the eight study groups with different supplementation in C57BL/6J mice. The boxes within each part of the figure represent one group, and the horizontal line inside them the mean of its abundance. Data are presented as log-transformed (log_10_) counts of bacterial 16 S rRNA gene copies

Microbiota analyses performed by 16s metagenomics revealed significant differences between the eight study groups (Fig. 6). Supplementation of mice with the three synbiotics reduced the abundance of Prevotellaceae and Sutterellaceae families compared with the HFS group (Fig. 6a,b). Moreover, in the case of Prevotellaceae, a synergistic effect was observed between pA1c, PC and BGC, as supplementation with the three synbiotics resulted in a much lower abundance of this family than supplementation with pA1c.

## Discussion

*C. elegans* is a small nematode that can be maintained at low cost and handled using standard in vitro techniques. This experimental model can be applied in the identification, screening and selection of probiotics, prebiotics and synbiotics, from a large number of combinations, and to better understand the mechanism of action of these compounds [31]. Nevertheless, the fact that *C. elegans* is a non-complex organism can be an obstacle because the targets and the underlying elucidated mechanisms may not be like those in animals and humans [32]. Therefore, as well as the in vivo study in *C. elegans*, we completed this work by testing the different preselected combinations in rodents with diet-induced obesity.

As *C. elegans* is a simple organism, its lipid metabolism is not very complex and is dependent on many factors, such as longevity [33], diet [34] or ageing [35]. Being a non-complex organism, it is not unusual that all the metabolic pathways of the worm are related and interconnected. A relationship has been reported between lipid metabolism and mitochondria [36], AMP-activated kinase [37], high-dietary carbohydrate intake [38] and glucose metabolism [39]. Previous works have established that glucose increases fat accumulation and upregulates the IIS, therefore reducing the expression of the downstream component of the IIS *daf-16* (the main gene involved in glucose metabolism) in *C. elegans* [12, 40, 41]. Moreover, we demonstrated that the IIS plays an important role in the mechanism of action of the probiotic, as well as in β-oxidation and fatty acid biosynthesis and degradation [12]. Hence, having shown the effect of the different combinations of synbiotic on fat accumulation, lifespan and health, we set out to examine whether the synbiotics could enhance the effect of the probiotic alone on the different metabolic pathways affected by pA1c itself. It is known that β-glucans have fat-reducing properties independently of *daf-16* [42]. Additionally, other groups have shown that the activity of these prebiotics are mediated by lipid metabolism pathways [43]. Thus, considering the previous results and the results of this work, we can hypothesise that the remaining fat-reducing effect of the synbiotic in NGMg is attributed to the action of β-glucans, since it is not reported that PC is capable of reducing fat accumulation and, as we have demonstrated in our previous study, the fat-reducing capacity of the probiotic is IIS-dependent and does not have activity in a *daf-16* null mutant [12].

Furthermore, our previous studies have reported that pA1c inhibits the high-glucose-induced nuclear translocation of *daf-16* by affecting the IIS. In normal conditions, *daf-16* remains in the cytosol of the cells of the worm but, when glucose is added to the medium, *daf-16* translocates to the nucleus of the cells of *C. elegans* as a defensive action from the worm to cope with the stress induced by glucose. In this study, we observed that in pA1c+PC+BGC-supplemented worms the glucose nuclear localisation of *daf-16* was reversed in a more effective way than in worms supplemented with pA1c alone. Although no studies have shown that PC and BGC are capable of modulating the location and the translocation of *daf-16*, we can assume a synbiotic effect in the translocation of *daf-16* between pA1c and these two bioactive compounds, due to the enhanced normalising activity observed vs pA1c alone.

With regard to fat and muscle gene expression, we suggest that the mechanism of action by which the probiotic and the synbiotics exert their hypoglycaemic and lipid-lowering effects is through the IIS. As a compensation for hyperglycaemia and insulin resistance, probiotic- and synbiotic-supplemented mice upregulated the expression of the insulin receptor gene *Insr* and the glucose transporters GLUT-1 and GLUT-4 in both fat and muscle tissues, by the overexpression of the coding genes *Glut-1* and *Glut-4*. This overexpression increases the uptake of glucose by muscle tissue and activated the PI3K/Akt-1 pathway [44] by upregulating *Akt-1* expression [45], which in turn leads to a decline in the expression levels of *Foxo-1* and *Pdk-4* [46] to counteract the excess of glucose and inhibit the insulin resistance. Taken together, our data demonstrate that the synbiotic combinations that include pA1c have blood glucose-regulating effects and alleviate insulin resistance by affecting the IIS (upregulating *Glut-1, Glut-4* in adipose and muscle tissue*,* and overexpressing *Akt-1* and *Insr* and downregulating *Foxo-1* and *Pdk-4* in muscle tissue), reducing inflammation (downregulating CRP and MCP-1 concentration), and promoting β-oxidation (overexpression of *Acox-1* and *Cpt-2*) in mice.

In recent years, different metagenomic analyses have shown that certain changes in the microbiota are closely related to the development of different human diseases [47, 48]. Regarding diabetes, several studies have established a strong correlation between dysbiosis of the intestinal microbiota and the emergence of diabetes complications, such as insulin resistance, decreased glucose tolerance and development of inflammation [49, 50]. One novel and promising approach to type 2 diabetes targets the modulation of gut microbiota with probiotics, prebiotics and synbiotics with the aim of counteracting the dysbiosis and restoring gut microbiota homeostasis. In this work, we have supplemented C57BL/6J mice with the different treatments and compared the changes occurring in the microbiota after each treatment with the microbiota of the diet and fructose-induced diabetes group (HFS).

Following supplementation of mice with PC and BGC, there was a decrease in the abundance of the families Eubacteriaceae, Christensenellaceae, Lachnospiraceae and Ruminococcaceae and an increase in Bifidobacteriaceae, Lactobacillaceae and Verrucomicrobiaceae. As well as *Asaccharobacter*, Eubacteriaceae have been found to be increased in people with depression [29]. Previous studies have shown that sodium butyrate alleviates diabetes-induced intestinal inflammation in mice by reducing the relative abundance of Christensenellaceae and modulating the microbiota [51]. This can explain the reduction in inflammation found in our hyperglycaemic and diabetic mice and may be one of the mechanisms of action of the synbiotic combinations by which they improved the inflammatory profile. Other works have demonstrated that resveratrol reduced obesity in high-fat-diet-fed mice via modulating the composition and metabolic function of the gut microbiota, reducing the abundance of the genera Lachnospiraceae [52]. This work has associated the Lachnospiraceae_NK4A136 group with obesity and other associated metabolic disorders (insulin resistance) and reported this bacterium as a discriminative feature of gut dysbiosis [53]. This could explain the fat-reducing properties found in *C. elegans* and confirmed by the reduction of visceral WAT observed in mice supplemented with the synbiotics. Moreover, it has been shown that women with gestational diabetes mellitus (GDM) presented enriched abundance of Lachnospiraceae in comparison with control pregnant women [54]. Further, a reduction in both Lachnospiraceae and Ruminococcaceae abundance has been associated with the metabolic improvements after treatment with liraglutide (an oral glucose-lowering drug) [55]. The decrease in the abundance of these families, together with the decrease in Eubacteriaceae, could explain the anti-inflammatory effect of synbiotics, in addition to the reduction in FBG and insulin resistance. On the other hand, we observed an important increase in the abundance of Bifidobacteriaceae, Lactobacillaceae and Verrucomicrobiaceae in all groups containing BGC, families negatively correlated with the appearance of diabetes and found to be decreased in individuals with diabetes [56–59], demonstrating the protective function of BGC against diabetes.

Differences in the abundance of genera following PC and BGC supplementation included a decrease in *Alloprevotella*, *Christensenella*, *Lachnospira*, *Odoribacter* and *Ruminococcus* among others, and an increase in *Akkermansia*, *Bifidobacterium* and *Lactobacillus*. According to Zhao et al, *Alloprevotella* is a harmful bacteria, and its abundance is found to be higher in high-fat-diet-fed mice [60], so the decrease in its abundance could be a way to prevent high-fat-diet-induced obesity, the metabolic syndrome and gut dysbiosis. A study in women with GDM demonstrated that *Christensenella* was associated with higher fasting plasma glucose concentrations [61], hence reduction of its abundance may be a good strategy to reduce glucose spikes and enhance glycaemic control. *Lachnospira* has been reported to be increased in individuals with diabetes who have retinopathy, compared with healthy individuals [62]. In addition, a study has reported that lower amounts of *Lachnospira* can ameliorate high-fat-diet-induced obesity in mice by stimulating the browning of the WAT and modulating gut microbiota [63]. This may explain the increase in brown fat and the reduction of visceral fat found in mice supplemented with the triple synbiotic. Other authors found that *Sanghuangporus vaninii* mixture ameliorated type 2 diabetes and altered intestinal microbiota in mice through reducing the abundance of *Odoribacter* [64]. Besides, other researchers have established that the genera *Ruminococcus* was positively correlated with type 2 diabetes, while *Bifidobacterium* and *Akkermansia* were negatively associated with type 2 diabetes [50], thus explaining the metagenomic results that we have obtained. Moreover, other studies support these affirmations and also confer glucose-lowering properties upon *Akkermansia*, *Bifidobacterium* and *Lactobacillus* [50, 58, 65–67].

In the case of Prevotellaceae, a synergistic effect was observed between pA1c, PC and BGC, as supplementation with the three synbiotics resulted in a much lower abundance of this family than supplementation with pA1c. Previous studies from our group, reporting obesity-related inflammatory status based on gut microbiota composition, have revealed that the family Prevotellaceae was more abundant in individuals with a higher inflammatory score and was associated with a proinflammatory phenotype [47]. Moreover, animal and human studies support a causal role of the family Prevotellaceae in promoting low-grade chronic systemic inflammation through the activation and dissemination of inflammatory mediators [68]. Furthermore, Prevotellaceae are associated with insulin resistance and low-grade inflammation in adipose tissue from morbidly obese individuals [69]. Additionally, studies in Brazilian and American populations indicate that the Sutterellaceae family could be a potential pathobiont with the ability to cause certain dysbiosis-related pathologies [70]. Therefore, a reduction in the abundance of these two families in the synbiotic-supplemented groups could explain the improvement in the observed inflammatory profile and the reversal of the HFS diet- and 10% fructose-induced dysbiosis.

The three synbiotics, especially pA1c+BGC and pA1c+PC+BGC, decreased the abundance of the genera *Bacteroides*, *Butyrivibrio*, *Gemmiger*, *Paraprevotella*, *Parasutterrella*, *Prevotella* and *Turicibacter*, while they increased the abundance of the genus *Pediococcus* in comparison with the HFS group. It has been shown that a high abundance of *Bacteroides* spp. is associated with type 2 diabetes risk in obese individuals [71]. This suggests that an aberrant gut microbiota-immune axis in obese people may drive or aggravate type 2 diabetes. Furthermore, *Bacteroides* was found to be positively correlated with glucose levels in women with GDM [72], implying that a reduction in *Bacteroides* abundance would be beneficial in these individuals. Moreover, it has been established that *Bacteroides* spp. and the genus *Butyrivibrio* were decreased in functional constipated people on an anti-inflammatory diet [73], indicating that the reduction of these two genera caused by an anti-inflammatory diet could mitigate intestinal inflammation. Other investigations have established that individuals with diabetes and diabetic cognitive impairment present a greater abundance of *Bacteroides*, *Gemmiger* and *Prevotella* genera [74]. A possible application of the synbiotics tested in this study could be the alleviation of cognitive disturbances related to diabetes since the synbiotics reduced the abundance of these genera compared with the HFS group. Moreover, *Gemmiger* has been shown to be related to hepatocellular ballooning, which in turn is linked to the development of type 2 diabetes [75]. Additionally, it has been demonstrated that the relative abundance of *Gemmiger* is positively correlated with increased liver enzymes and overweight status [76]. Hence, this data demonstrate that individuals with a high abundance of *Gemmiger* are more likely to have increased liver enzymes and are more likely to be overweight. The *Paraprevotella* genera have been shown to play an important role in the development of both GDM and type 2 diabetes. *Paraprevotella* was shown to be increased in the placentas of women with GDM vs control individuals [77], and in those who went on to develop type 2 diabetes compared with matched controls [78]. A recent translational human study of the European cross-sectional FoCus cohort established that *Parasutterella* was positively associated with BMI, body weight gain and type 2 diabetes independently of the reduced microbiome α/β diversity and low-grade inflammation commonly found in obesity [79]. The same study demonstrated that the metabolite l-cysteine showed the strongest reduction in individuals with high *Parasutterella* abundance and proved that *Parasutterella* is a high l-cysteine consumer. This is very interesting since l-cysteine is known to improve blood glucose levels in rodents [80, 81]. Therefore, reduced *Parasutterella* abundance in the synbiotic-supplemented groups may explain the improvements in blood glucose, visceral fat, insulin resistance, β-oxidation and fatty acid biosynthesis that we observed in both *C. elegans* and mice. It has been reported that the relative abundance of *Prevotella* and *Alloprevotella* is significantly higher in individuals with type 2 diabetes [82]. Consequently, reduced abundance of these two genera would lead to an improvement in the glycaemic profile of diabetic individuals. A study carried out in obese diabetic mice reported that in comparison with the non-diabetic group, *Turicibacter* was only present in the faeces of diabetic mice [83], suggesting that the reduction of this microorganism would be a good strategy to revert the complications originating from type 2 diabetes and obesity.

In the recent years, various studies have suggested the genus *Pediococcus* and the species *Pediococcus acidilactici* to be potential probiotics preventing hyperglycaemia and diabetes. High abundance of this bacterium is negatively correlated with glucose levels and diabetes development [12–14, 84, 85]. A significant increase in *Pediococcus* (Fig. 6h) and *Pediococcus acidilactici* (Fig. 6k) was detected in the three synbiotic-supplemented groups compared with the HFS group and pA1c group. It can be observed that control and HFS groups hardly had any *Pediococcus* and that the abundance was much higher in the synbiotic-supplemented groups, even more so than with the probiotic alone, due to the synergy between the components. So, we can conclude that PC and BGC help *Pediococcus acidilactici* colonisation and settling in the intestine.

**Fig. 7:**
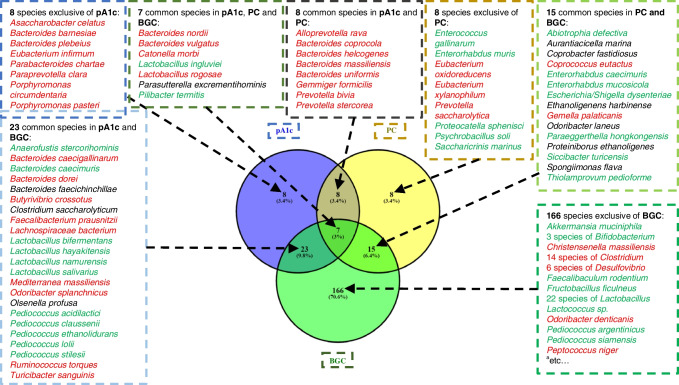
Venn diagram showing the species that are statistically associated with and shared between the three supplemented variables pA1c (blue), PC (yellow) and BGC (green). Species associated with higher levels of the corresponding supplemented variables, when compared with HFS group, appear in green font. Species associated with lower levels of the corresponding supplemented variables, when compared with HFS group, appear in red font. Species in which the synbiotic components present a different pattern of abundance (one showing increased abundance and the other showing decreased abundance), when compared with the HFS group, appear in black font. ^a^All species associated with the BGC group are listed in ESM Tables 6, 7 and 8

Having investigated the differences between families and genera in the supplementation groups, we then turned our attention to the species that were statistically associated and shared between the three supplemented variables pA1c, PC and BGC (Fig. 7). We will only discuss the groups in which the supplementation contained pA1c, as they are the most interesting ones. Eight species are exclusive of pA1c (all have reduced abundances compared with the HFS group), the following being the most remarkable: *Asaccharobacter celatus*, increased in people with depression [29]; *Bacteroides plebeius*, increased in individuals with diabetic kidney disease [86] and in diabetic obese people [87]; and *Paraprevotella clara*, *Porphyromonas circumdentaria* and *Porphyromonas pasteri*, considered to be opportunistic pathogens [88–90].

Furthermore, there are eight species common to pA1c and PC groups, that, as for pA1c, all exhibit lower abundance compared with the HFS group. *Alloprevotella rava* is a well-known periodontitis-inducing pathogen that can also be involved in localised diabetes complications [91]. Therefore, glucose-lowering treatments could reverse the microbiota of the saliva and hence improve the localised conditions of diabetic individuals with periodontitis. As stated above, a high abundance of *Bacteroides* spp., *Gemmiger formicilis* and *Prevotella stercorea* could be associated with diabetes risk in obese individuals, intestinal inflammation and diabetic cognitive impairment [71–75, 82]. In addition, a high abundance of *Prevotella bivia* seems to be associated with endocarditis [92], since it is a pathogenic bacterium.

Moreover, there are 23 species common to the pA1c and BGC groups. The main finding was the increase in many species of *Lactobacillus* spp. and *Pediococcus* spp., especially *Lactobacillus salivarius* and *Pediococcus acidilactici*. Focusing on the five species of *Pediococcus spp.* that are common to pA1c and BGC, shown in ESM Fig. 2, we can observe that all these species follow the same pattern of abundance. This leads us to suspect that all the species may actually be the one we used in the supplementation (*Pediococcus acidilactici*). This assumption is supported by several studies demonstrating the nonspecificity of 16s rRNA to detect, identify and separate different LAB species, such as *Lactobacillus* and *Pediococcus* [93, 94]. Furthermore, a similarity study between *Pediococcus lolii* and *Pediococcus acidilactici* revealed, by using the pair-wise similarity matrix, that *Pediococcus lolii* has 97.7% sequence similarity with *Pediococcus acidilactici*. The high degree of similarity between both strains indicates that *Pediococcus lolii* is a strain of *Pediococcus acidilactici* [95]. Numerous studies support the use of *Lactobacillus salivarius* and *Pediococcus acidilactici* as probiotics, since they are able to lower blood glucose levels, reduce proinflammatory cytokines and attenuate liver damage in individuals with type 1 and type 2 diabetes [12–14, 85, 96–98]. In addition, a decline in the relative abundance of *Butyrivibrio crossotus*, *Lachnospiraceae bacterium*, *Ruminococcus torques* and *Turicibacter sanguinis* among others can be observed. As commented before, a reduction in *Butyrivibrio crossotus* abundance helps to mitigate intestinal inflammation in individuals with constipation [73]. Besides, it has been demonstrated that intestinal colonisation by *Lachnospiraceae bacterium* and *Ruminococcus torques* contributes to the development of diabetes in obese mice [99], and confirms the observations made with the genera *Butyrivibrio* and *Ruminococcus*. As already mentioned, a study performed in obese diabetic mice demonstrated that, compared with the non-diabetic group, *Turicibacter* spp. are present in diabetic mice faeces and may play an important role in diabetes metabolism and development [83].

Finally, Fig. 7 shows that the pA1c, PC and BGC groups had seven species in common. High intestinal colonisation with *Bacteroides vulgatus* is found to be related to insulin resistance and low-grade inflammation in individuals with type 2 diabetes [100]. *Catonella morbi* has been associated with occasional infections [101]. On the other hand, a high abundance of *Lactobacillus ingluviei* is beneficial, as it exerts an anti-inflammatory effect by modulating cytokine profiles [102].

### Conclusions

In conclusion, we have shown a synergistic effect of the combination of pA1c, PC and BGC on the reduction of fat accumulation, ROS levels and ageing, and on the increase in lifespan, in comparison with no treatment and pA1c treatment in *C. elegans*. Besides, we have demonstrated that the molecular mechanisms by which the synbiotics exert their effects are modulation of the IIS and activation of mitochondrial and peroxisomal fatty acid degradation.

Furthermore, we have corroborated the findings found in *C. elegans* in mice, in which we found that the synbiotic-containing groups exhibited reduction in FBG, attenuation of postprandial hyperglycaemia following an i.p. glucose load, reduction, normalisation and maintenance of blood glucose levels, alleviation of insulin resistance, normalisation of serum and intrahepatic TG content, reduction of liver damage and inflammation, and reduction of visceral fat when compared with the HFS group. Moreover, we revealed that the main metabolic pathways by which the synbiotics are effective are the modulation of the glucose metabolism as follows: overexpression of *Glut-1* and *Glut-4* in adipose and muscle tissue, by the upregulation of *Akt-1* and *Insr* in muscle tissue; increase in the abundance of Bifidobacteriaceae, Lactobacillaceae, Verrucomicrobiaceae, *Bifidobacterium*, *Lactobacillus*, *Akkermansia*, *Pediococcus*, *Pediococcus acidilactici* and *Lactobacillus salivarius*; downregulation of *Foxo-1* and *Pdk-4* in muscle tissue; reduction in the abundance of Christensenellaceae, Lachnospiraceae, Ruminococcaceae, *Christensenella*, *Odoribacter*, *Ruminococcus*, *Bacteroides*, *Gemmiger*, *Paraprevotella*, *Parasutterella*, *Prevotella*, *Turicibacter*, *Lachnospiraceae bacterium*, *Ruminococcus torques*, *Turicibacter sanguinis*, *Bacteroides vulgatus*; upregulation of β-oxidation by the overexpression of *Acox-1*, *Cpt-2* and *Adipoq* in adipose tissue and reduction in the abundance of *Saccharofermentans*, *Alloprevotella* and *Parasutterella*; and decrease in inflammation by reducing the proinflammatory molecules CRP and MCP-1, reducing the abundance of Prevotellaceae*, Bacteroides*, *Butyrivibrio*, *Gemmiger*, *Butyrivibrio crossotus* and *Gemmiger formiculis*, and increasing the abundance of *Lactobacillus ingluviei*.

Although knowledge of the use of biotics against diabetes is still rather limited and there is a great variety among the data from studies, the absence of side effects and the promising results described in this research work should encourage further studies on gut microbiota modulation by synbiotics for the prevention and treatment of diabetes. More in-depth studies of the interaction between the supplemented synbiotics and the host microbiota are needed to understand the biomolecular mechanisms by which pA1c-containing synbiotics exert their diabetes-alleviatory effect.

Our results suggest that supplementation with synbiotics containing pA1c significantly enhance the carbohydrate and lipid metabolism of *C. elegans* and mice. Therefore, the combination of pA1c with PC and BGC could be considered as a potential synbiotic for the prevention and treatment of type 2 diabetes and its complications such as the metabolic syndrome, obesity, insulin resistance or chronic inflammation. Besides, our data highlight the use of *C. elegans* as an appropriate in vivo model, together with the 16s rRNA metagenomic analyses for the study of the mechanisms underlying these diseases.

### Supplementary Information

Below is the link to the electronic supplementary material.

Supplementary file1 (PDF 579 KB)

## Data Availability

All data supporting the findings of this study are available within the paper and the ESM.

## Abbreviations

AIP: Atherogenic index of plasma
ALT: Alanine aminotransferase
AST: Aspartate aminotransferase
BAT: Brown adipose tissue
BGC: Oat β-glucans
CFU: Colony-forming units
CRP: C-Reactive protein
DHE: Dihydroethidium
FBG: Fasting blood glucose
GDM: Gestational diabetes mellitus
HDL-Chol: HDL-cholesterol
HFS: High-fat/high-sucrose
IIS: Insulin signalling pathway
LAB: Lactic acid bacteria
MCP-1: Monocyte chemotactic protein-1
NGM: Nematode growth medium
NGMg: Glucose-loaded NGM
ORO: Oil Red O
OTU: Operational taxonomic unit
pA1c: *Pediococcus acidilactici* CECT9879
PC: Chromium picolinate
qPCR: Quantitative real-time PCR
ROS: Reactive oxygen species
SNK: Student–Newman–Keuls
TG: Triacylglycerol
TyG: Triglycerides and glucose
WAT: White adipose tissue
