# Supplementary material for: Glucose-lowering effects of a synbiotic combination containing Pediococcus acidilactici in C. elegans and mice
Source: Diabetologia. 2023 Aug 16;66(11):2117–38. doi: 10.1007/s00125-023-05981-w (PMC10542285; doi:10.1007/s00125-023-05981-w)
Supplement: Supplementary file 1 — Supplementary file1 (PDF 579 KB) [file 125_2023_5981_MOESM1_ESM.pdf]

ESM TABLE 1

ESM Table 1 | Significant gut microbial changes in C57BL/6J mice due to the supplementation of pA1c, PC or BGC.

pA1c		PC		BGC	
**Families**	**Genera**	**Families**	**Genera**	**Families**	**Genera**
Oceanospirillaceae	*Asaccharobacter*	Chromatiaceae	*Acetobacteroides*	Bacteroidaceae	*Akkermansia*
Saprospiraceae	*Lactococcus*	Enterobacteriaceae	*Alloprevotella*	Bifidobacteriaceae	*Bifidobacterium*
	Lysinibacillus	Enterococcaceae	*Catabacter*	Christensenellaceae	*Christensenella*
	Saccharofermentans	Eubacteriaceae	*Coprobacter*	Clostridiaceae	*Clostridium*
		Planococcaceae	*Enterococcus*	Flavobacteriaceae	*Faecalicoccus*
			Enterorhabdus	Lachnospiraceae	*Fructobacillus*
			Eubacterium	Lactobacillaceae	*Lachnospira*
			Fibrobacter	Proteinivoraceae	*Lactobacillus*
			Kosakonia	Ruminococcaceae	*Odoribacter*
			Paenisporosarcina	Verrucomicrobiaceae	*Proteinivorax*
			Planococcaceae	etc...	*Rikenella*
			Pseudopedobacter		*Ruminococcus*
			Serratia		etc...

ESM Table 1. Significant gut microbial changes in family and genera in C57BL/6J mice due to the supplementation of pA1c, PC or BGC. Families and genera associated with higher levels of the corresponding supplemented variables in comparison with HFS group are written in green font. Families and genera associated with lower levels of the corresponding supplemented variables in comparison with HFS group are written in red font. Each section of ESM Table 1 includes all the groups that received that supplement, and all are compared with the HFS group: the pA1c section includes all the groups that were supplemented with pA1c (pA1c group, pA1c+PC, pA1c+BGC and pA1c+PC+BGC); the PC section includes the PC group, pA1c+PC group and pA1c+PC+BGC group; and the BGC section included the BGC group, pA1c+BGC group and pA1c+PC+BGC group. Families and genera listed in each section are common to all the groups that were supplemented with that compound, and are restricted to only one section, which means that the same family or genera can only appear in one section (the supplemented group with the highest abundance of the microorganism).

ESM TABLE 2

ESM Table 2 | Completed list of significant gut microbial changes in families in C57BL/6J mice due to the supplementation of BGC compared with HFS

Acholeplasmataceae	Catabacteriaceae	Defluviitaleaceae	Marinilabiliaceae	Ruminococcaceae
Acidaminococcaceae	Chitinophagaceae	Deinococcaceae	Methylobacteriaceae	Shewanellaceae
Acidimicrobiaceae	Chlorobiaceae	Elusimicrobiaceae	Microbacteriaceae	Sinobacteraceae
Actinomycetaceae	Christensenellaceae	Enterobacteriaceae	Mycobacteriaceae	Sphingobacteriaceae
Aerococcaceae	Clostridiaceae_1	Entomoplasmataceae	Nautiliaceae	Sphingomonadaceae
Alcanivoracaceae	Clostridiaceae_2	Erysipelotrichaceae	Nocardiaceae	Spirochaetaceae
Anaeroplasmataceae	Clostridiaceae_3	Family_II	Peptococcaceae_1	Staphylococcaceae
Bacillaceae_2	Clostridiaceae_4	Family_XIII	Peptoniphilaceae	Streptomycetaceae
Bacillales_incertae_sedis	Clostridiales_incertae_sedis	Flavobacteriaceae	Peptostreptococcaceae	Syntrophomonadaceae
Bacillales_Incertae_Sedis_XI	Clostridiales_Incertae_Sedis_XII	Gracilibacteraceae	Piscirickettsiaceae	Thermoanaerobacteraceae
Bacteroidaceae	Clostridiales_Incertae_Sedis_XIII	Halobacteroidaceae	Prolixibacteraceae	Thermodesulfobiaceae
Bifidobacteriaceae	Clostridiales_Incertae_Sedis_XIV	Ktedonobacteraceae	Proteinivoraceae	Unclassified
Caldilineaceae	Colwelliaceae	Lachnospiraceae	Rhodospirillaceae	Verrucomicrobiaceae
Carnobacteriaceae	Cryomorphaceae	Lactobacillaceae	Rikenellaceae	Xanthomonadaceae

ESM Table 2. Completed list of significant gut microbial changes in families in C57BL/6J mice due to the supplementation of BGC. Families associated with higher levels of the corresponding supplemented variables in comparison with HFS group are in green cells. Families associated with lower levels of the corresponding supplemented variables in comparison with HFS group are in red cells.

ESM TABLE 3

ESM Table 3 | Completed list of significant gut microbial changes in genera in C57BL/6J mice due to the supplementation of BGC compared with HFS

Abiotrophia	*Anaerobranca*	*Bilophila*	*Clostridium_sensu_stricto*	*Desulfocurvus*
Acetatifactor	*Anaerofilum*	*Blautia*	*Clostridium_XlVa*	*Dethiosulfovibrio*
Acetitomaculum	*Anaerofustis*	*Brockia*	*Clostridium_XlVb*	*Dialister*
Acetivibrio	*Anaeroglobus*	*Bulleidia*	*Coenonia*	*Dorea*
Acetoanaerobium	*Anaeroplasma*	*Butyrivibrio*	*Cohnella*	*Eggerthella*
Acholeplasma	*Anaerosphaera*	*Caldicellulosiruptor*	*Collinsella*	*Eisenbergiella*
Actinomyces	*Anaerostipes*	*Caloranaerobacter*	*Colwellia*	*Elusimicrobium*
Agromyces	*Anaerotruncus*	*Capnocytophaga*	*Coprococcus*	*Erysipelothrix*
Akkermansia	*Anoxybacillus*	*Carboxylicivirga*	*Coprothermobacter*	*Erysipelotrichaceae_incertae_sedis*
Alicyclobacillus	*Aquimarina*	*Catenisphaera*	*Cronobacter*	*Escherichia/Shigella*
Alistipes	*Atopobium*	*Catonella*	*Cyclobacterium*	*Ethanoligenens*
Alkalibacterium	*Aureispira*	*Cellulosilyticum*	*Defluviitalea*	*Facklamia*
Alkaliphilus	*Bacillus*	*Christensenella*	*Deinococcus*	*Faecalibacterium*
Allobaculum	*Bifidobacterium*	*Citrobacter*	*Desemzia*	*Faecalicoccus*

ESM Table 3. Completed list of significant gut microbial changes in genera in C57BL/6J mice due to the supplementation of BGC. Genera associated with higher levels of the corresponding supplemented variables in comparison with HFS group are in green cells. Genera associated with lower levels of the corresponding supplemented variables in comparison with HFS group are in red cells.

ESM TABLE 4

ESM Table 4 | Completed list of significant gut microbial changes in genera in C57BL/6J mice due to the supplementation of BGC compared with HFS

Flavobacterium	*Holdemanella*	*Marinifilum*	*Oligella*
Flavonifractor	*Hydrogenoanaerobacterium*	*Marvinbryantia*	*Olsenella*
Fusibacter	*Ilumatobacter*	*Meniscus*	*Oribacterium*
Fusicatenibacter	*Intestinimonas*	*Mesoplasma*	*Oscillibacter*
Gemella	*Kangiella*	*Methylobacterium*	*Papillibacter*
Gordonibacter	*Ktedonobacter*	*Methylogaea*	*Paraeggerthella*
Gp4	*Lachnoanaerobaculum*	*Moryella*	*Paraprevotella*
GpI	*Lachnospira*	*Mucinivorans*	*Parvibacter*
GpIIa	*Lachnospiracea_incertae_sedis*	*Murimonas*	*Pediococcus*
GpXIII	*Lactobacillus*	*Mycobacterium*	*Pedobacter*
Guggenheimella	*Lactonifactor*	*Mycoplasma*	*Pelagicoccus*
Halanaerobium	*Latescibacteria_genera_incertae_sedis*	*Natranaerovirga*	*Peptococcus*
Halomonas	*Lawsonia*	*Nevskia*	*Peptostreptococcaceae_incertae_sedis*
Hespellia	*Lutispora*	*Odoribacter*	*Photorhabdus*

ESM Table 4. Completed list of significant gut microbial changes in genera in C57BL/6J mice due to the supplementation of BGC. Genera associated with higher levels of the corresponding supplemented variables in comparison with HFS group are in green cells. Genera associated with lower levels of the corresponding supplemented variables in comparison with HFS group are in red cells.

ESM TABLE 5

ESM Table 5 | Completed list of significant gut microbial changes in genera in C57BL/6J mice due to the supplementation of BGC compared with HFS

Pilibacter	*Saccharicrinis*	*Stomatobaculum*
Plasticicumulans	*Scardovia*	*Subdivision3_genera_incertae_sedis*
Polaribacter	*Selenihalanaerobacter*	*Syntrophococcus*
Porphyromonas	*Shewanella*	*Tetragenococcus*
Prosthecochloris	*Shuttleworthia*	*Treponema*
Proteiniborus	*Siccibacter*	*Turicibacter*
Proteinivorax	*Slackia*	*Unclassified*
Pseudoflavonifractor	*Sphingobacterium*	*Ureibacillus*
Pseudomonas	*Sphingomonas*	*Vallitalea*
Rikenella	*Spiroplasma*	*Vibrio*
Roseburia	*Spongiimonas*	*Weissella*
Ruminococcus	*Sporobacter*	
Ruminococcus2	*Sporosarcina*	
Saccharibacteria_genera_incertae_sedis	*Staphylococcus*	

ESM Table 5. Completed list of significant gut microbial changes in genera in C57BL/6J mice due to the supplementation of BGC. Genera associated with higher levels of the corresponding supplemented variables in comparison with HFS group are in green cells. Genera associated with lower levels of the corresponding supplemented variables in comparison with HFS group are in red cells.

ESM TABLE 6

ESM Table 6 | Completed list of significant gut microbial changes in species in C57BL/6J mice due to the supplementation of BGC compared with HFS

Abiotrophia_defectiva(D50541)	*Aureibacter_tunicatorum(AB572584)*	*Caproiciproducens_galactitolivorans(NR_145929.1)*	*Collinsella_tanakaei(AB490807)*
Acetatifactor_muris(HM989805)	*Bacteroides_caecigallinarum(NR_145844.1)*	*Catonella_morbi(X87151)*	*Coprobacter_fastidiosus(JN703378)*
Acetivibrio_ethanolgignens(FR749897)	*Bacteroides_caecimuris(NR_144606.1)*	*Christensenella_massiliensis(NR_144742.1)*	*Coprococcus_comes(EF031542)*
Acutalibacter_muris(NR_144605.1)	*Bacteroides_dorei(AB242142)*	*Clostridium_aldenense(DQ279736)*	*Coprococcus_eutactus(EF031543)*
Agromyces_rhizospherae(AB023357)	*Bacteroides_faecichinchillae(AB574480)*	*Clostridium_aminophilum(L04165)*	*Cuneatibacter_caecimuris(NR_144608.1)*
Akkermansia_muciniphila(AY271254)	*Bacteroides_intestinalis(AB214328)*	*Clostridium_asparagiforme(AJ582080)*	*Cytophaga_sp.(KF620113)*
Alistipes_finegoldii(AY643083)	*Bacteroides_nordii(AY608697)*	*Clostridium_citroniae(DQ279737)*	*Desemzia_incerta(Y17300)*
Alistipes_obesi(NR_133025.1)	*Bacteroides_salanitronis(AB253731)*	*Clostridium_innocuum(NR_029164.1)*	*Desulfovibrio_aespoeensis(X95230)*
Alistipes_senegalensis(NR_118219.1)	*Bacteroides_vulgatus(CP000139)*	*Clostridium_lactatifermentans(AY033434)*	*Desulfovibrio_arcticus(DQ296030)*
Allobaculum_sp.(AB537978)	*Barnesiella_intestinihominis(AB370251)*	*Clostridium_methylpentosum(Y18181)*	*Desulfovibrio_cuneatus(X99501)*
Allobaculum_stercoricanis(AJ417075)	*Barnesiella_viscericola(AB267809)*	*Clostridium_polysaccharolyticum(X77839)*	*Desulfovibrio_gabonensis(U31080)*
Anaerobium_acetethylicum(NR_137405.1)	*Bifidobacterium_animalis(D86185)*	*Clostridium_populeti(X71853)*	*Desulfovibrio_legallii(FJ225426)*
Anaerofilum_pentosovorans(X97852)	*Bifidobacterium_bohemicum(FJ858736)*	*Clostridium_propionicum(X77841)*	*Desulfovibrio_piger(AF192152)*
Anaerofustis_stercorihominis(AJ518871)	*Bifidobacterium_magnum(D86193)*	*Clostridium_saccharolyticum(Y18185)*	*Desulfovibrio_vietnamensis(X93994)*
Anaeroplasma_abactoclasticum(M25050)	*Bilophila_wadsworthia(AJ867049)*	*Clostridium_scindens(AF262238)*	*Desulfovibrio_vulgaris(AF418179)*
Anaerostipes_sp.(JX273468)	*Blautia_hydrogenotrophica(X95624)*	*Clostridium_sufflavum(AB267266)*	*Dorea_formicigenerans(L34619)*
Anaerotruncus_colihominis(AJ315980)	*Breznakia_blatticola(NR_146686.1)*	*Clostridium_vincentii(X97432)*	*Dorea_longicatena(AJ132842)*
Anaerotruncus_rubiinfantis(NR_147398.1)	*Breznakia_pachnodae(NR_146687.1)*	*Clostridium_viride(X81125)*	*Drancourtella_massiliensis(NR_144722.1)*
Arthrobacter_phenanthrenivorans(AM176541)	*Bulleidia_extructa(AF220064)*	*Clostridium_xylanovorans(AF116920)*	*Eggerthella_sinensis(AY321958)*
Aurantiacicella_marina(NR_147742.1)	*Butyrivibrio_crossotus(FR733670)*	*Coenonia_anatina(Y17612)*	*Eisenbergiella_tayi(KF814111)*

ESM Table 6. Completed list of significant gut microbial changes in species in C57BL/6J mice due to the supplementation of BGC. Species associated with higher levels of the corresponding supplemented variables in comparison with HFS group are in green cells. Species associated with lower levels of the corresponding supplemented variables in comparison with HFS group are in red cells.

ESM TABLE 7

ESM Table 7 | Completed list of significant gut microbial changes in species in C57BL/6J mice due to the supplementation of BGC compared with HFS

Elusimicrobium_minutum(AM490846)	*Gabonibacter_massiliensis(NR_146820.1)*	*Lactobacillus_hamsteri(AJ306298)*	*Lactococcus_sp.(AB699722)*
Enterococcus_mundtii(AF061013)	*Gemella_palaticanis(Y17280)*	*Lactobacillus_hayakitensis(AB267406)*	*Lactonifactor_longoviformis(DQ100449)*
Enterorhabdus_caecimuris(DQ789120)	*Gemella_sanguinis(Y13364)*	*Lactobacillus_hominis(FR681902)*	*Lutispora_thermophila(AB186360)*
Enterorhabdus_mucosicola(AM747811)	*Gordonibacter_pamelaeae(AM886059)*	*Lactobacillus_iners(Y16329)*	*Mediterranea_massiliensis(NR_144747.1)*
Escherichia/Shigella_dysenteriae(X96966)	*Guggenheimella_bovis(AY272039)*	*Lactobacillus_ingluviei(AF333975)*	*Meniscus_glaucopis(GU269545)*
Ethanoligenens_harbinense(AY295777)	*Hespellia_porcina(AF445239)*	*Lactobacillus_insicii(NR_147740.1)*	*Methylogaea_oryzae(EU672873)*
Eubacterium_biforme(M59230)	*Hydrogenoanaerobacterium_saccharovorans(EU158190)*	*Lactobacillus_intestinalis(AJ306299)*	*Mordavella_massiliensis(NR_147406.1)*
Eubacterium_dolichum(L34682)	*Ktedonobacter_racemifer(AM180156)*	*Lactobacillus_mellis(JX099545)*	*Mucinivorans_hirudinis(HG934468)*
Eubacterium_eligens(L34420)	*Lachnoanaerobaculum_umeaense(FJ796700)*	*Lactobacillus_murinus(AJ621554)*	*Muribaculum_intestinale(NR_144616.1)*
Eubacterium_hallii(L34621)	*Lachnoclostridium_pacaense(NR_147396.1)*	*Lactobacillus_namurensis(AM259118)*	*Muricomes_intestini(NR_144617.1)*
Eubacterium_pyruvativorans(AJ310135)	*Lachnospira_multipara(FR733699)*	*Lactobacillus_reuteri(NR_119069.1)*	*Mycobacterium_chitae(X55603)*
Eubacterium_siraeum(L34625)	*Lachnospiraceae_bacterium(KC311366)*	*Lactobacillus_rogosae(NR_104836.1)*	*Negativibacillus_massiliensis(NR_147378.1)*
Facklamia_ignava(Y15716)	*Lactobacillus_algidus(AB033209)*	*Lactobacillus_ruminis(AB326354)*	*Odoribacter_denticanis(AY560020)*
Faecalibacterium_prausnitzii(AJ413954)	*Lactobacillus_amylolyticus(Y17361)*	*Lactobacillus_saerimneri(AY255802)*	*Odoribacter_laneus(AB490805)*
Faecalibaculum_rodentium(NR_146011.1)	*Lactobacillus_amylotrophicus(AM236149)*	*Lactobacillus_salivarius(AF089108)*	*Odoribacter_splanchnicus(L16496)*
Fenollaria_massiliensis(NR_133038.1)	*Lactobacillus_apis(KF386017)*	*Lactobacillus_sicerae(HG794492)*	*Olegusella_massiliensis(NR_146815.1)*
Flexithrix_dorotheae(AB078077)	*Lactobacillus_bifermentans(JN175330)*	*Lactobacillus_sp.(HQ851022)*	*Oligella_ureolytica(AJ251912)*
Flintibacter_butyricus(NR_144611.1)	*Lactobacillus_concavus(AY683322)*	*Lactobacillus_sp.(KJ078643)*	*Oligella_urethralis(AF133538)*
Fructobacillus_ficulneus(AF360736)	*Lactobacillus_equi(AB048833)*	*Lactobacillus_taiwanensis(EU487512)*	*Olsenella_profusa(AF292374)*
Fusicatenibacter_saccharivorans(AB698910)	*Lactobacillus_faecis(AB812750)*	*Lactobacillus_tucceti(AJ576006)*	*Olsenella_sp.(JX905358)*

ESM Table 7. Completed list of significant gut microbial changes in species in C57BL/6J mice due to the supplementation of BGC. Species associated with higher levels of the corresponding supplemented variables in comparison with HFS group are in green cells. Species associated with lower levels of the corresponding supplemented variables in comparison with HFS group are in red cells.

ESM TABLE 8

ESM Table 8 | Completed list of significant gut microbial changes in species in C57BL/6J mice due to the supplementation of BGC compared with HFS

Olsenella_uli(AF292373)	Proteiniborus_ethanoligenes(EF116488)	Sporobacter_termitidis(Z49863)
Olsenella_umbonata(FN178463)	Proteinivorax_tanatarense(JQ904541)	Stomatobaculum_longum(HM120209)
Oscillibacter_sp.(JF750939)	Pseudoflavonifractor_phocaeensis(NR_147370.1)	Streptococcus_danieliae(GQ456229)
Papillibacter_cinnamivorans(AF167711)	Raoultibacter_massiliensis(NR_144751.1)	Syntrophococcus_sucromutans(AF202264)
Parabacteroides_merdae(AB238928)	Rikenella_microfusus(L16498)	Thermovirga_lienii(DQ071273)
Paraeggerthella_hongkongensis(AY288517)	Roseburia_faecis(AY305310)	Thiolamprovum_pedioforme(Y12297)
Parasutterella_excrementihominis(AB370250)	Roseburia_intestinalis(AJ312385)	Turicibacter_sanguinis(AF349724)
Parvibacter_caecicola(GQ456228)	Ruminococcus_albus(L76598)	uncultured_Acidobacteria(Z95729)
Pediococcus_acidilactici(AJ305320)	Ruminococcus_faecis(FJ611794)	Ureibacillus_sp.(JX274433)
Pediococcus_argentinicus(AM709786)	Ruminococcus_flavefaciens(L76603)	Vallitalea_pronyensis(KC876639)
Pediococcus_claussenii(AJ621555)	Ruminococcus_gauvreauii(EF529620)	Vibrio_mediterranei(X74710)
Pediococcus_ethanolidurans(AY956789)	Ruminococcus_gnavus(X94967)	
Pediococcus_lolii(AB362985)	Ruminococcus_torques(NR_115502.1)	
Pediococcus_siamensis(AB258357)	Scardovia_wiggsiae(AY278626)	
Pediococcus_stilesii(AJ973157)	Selenihalanaerobacter_shriftii(AF310247)	
Peptococcus_niger(X55797)	Shuttleworthia_satelles(AF399956)	
Phocea_massiliensis(NR_144748.1)	Siccibacter_turicensis(DQ273681)	
Pilibacter_termitis(AY533171)	Sphingobacterium_bambusae(GQ339910)	
Plasticicumulans_lactativorans(JN565849)	Spongiiferula_fulva(NR_146705.1)	
Prevotella_copri(AB064923)	Spongiimonas_flava(AB742039)	

ESM Table 8. Completed list of significant gut microbial changes in species in C57BL/6J mice due to the supplementation of BGC. Species associated with higher levels of the corresponding supplemented variables in comparison with HFS group are in green cells. Species associated with lower levels of the corresponding supplemented variables in comparison with HFS group are in red cells.

ESM FIG. 1.

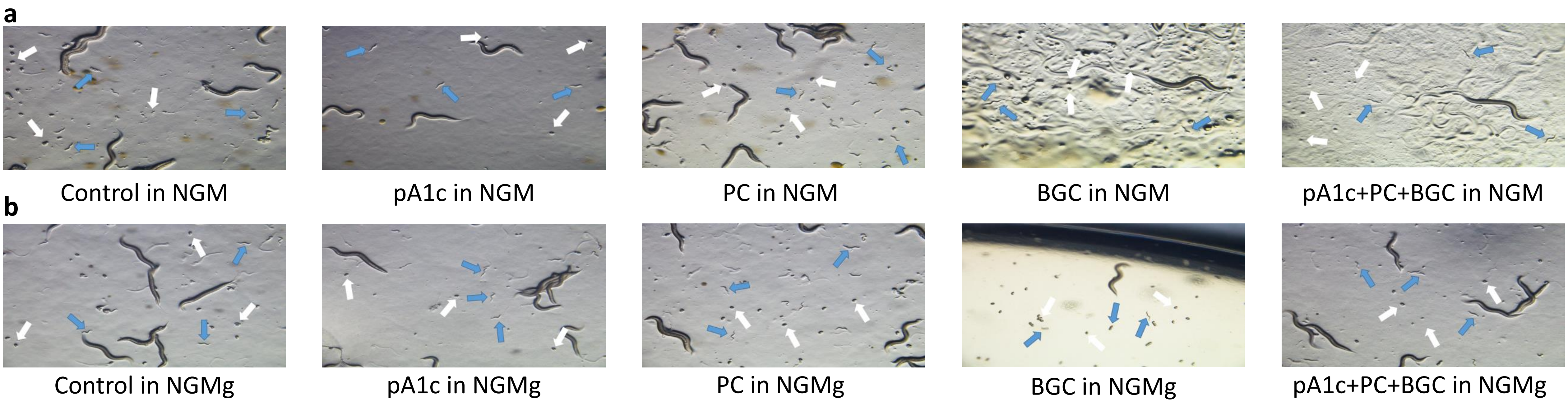

ESM Fig. 1. Microscope observation of the presence of eggs (white arrows) and stadium L1 larvae (blue arrows) in water-, pA1c-, PC-, BGC- and pA1c+PC+BGC-supplemented plates in NGM (**a**) and NGMg (**b**).

ESM FIG. 2.

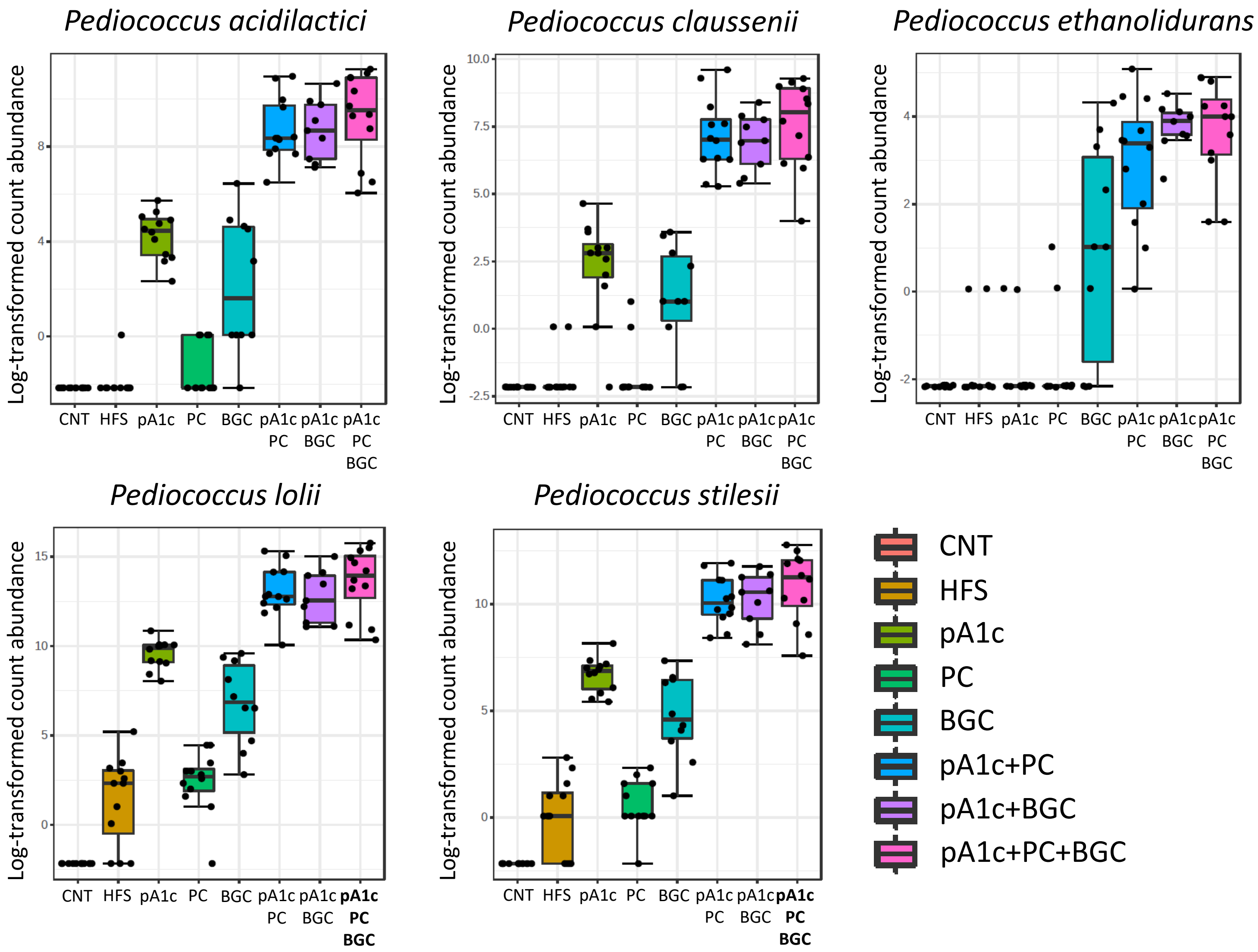

ESM Fig. 2. *Pediococcus* species differential between the eight groups of the study with different supplementation in C57BL/6J mice. Data were log-transformed counts of bacterial 16 S rRNA gene copies.
